# Ion‐Regulating Membranes with Surface‐Enriched Charge Networks Enabling Stable Zinc‐Manganese Flow Batteries

**DOI:** 10.1002/adma.202517473

**Published:** 2025-12-12

**Authors:** Jine Wu, Jiafeng Lei, Yi‐Chun Lu

**Affiliations:** ^1^ Department of Mechanical and Automation Engineering Electrochemical Energy and Interfaces Laboratory The Chinese University of Hong Kong Hong Kong SAR China

**Keywords:** energy storage, ion transport mechanism, membrane design, zinc‐based flow batteries

## Abstract

Zinc‐based flow batteries are promising for sustainable energy storage owing to their high energy density and eco‐friendliness. When coupling with Mn^2+^/MnO_2_ posolyte, the zinc‐manganese flow batteries promise an ultra‐low electrolyte cost (0.0039 $ Ah^−1^). However, their practical application is limited by low areal capacity (<20 mAh cm^−2^) and poor lifespan (<100 cycles with accumulated capacity < 2000 mAh cm^−2^), associated with proton crossover and zinc dendrite formation. To address the two bottlenecks, an ion‐regulating membrane with surface‐enriched positive charges of Zn^2+^ crosslinked networks is proposed. The enriched‐charged networks amplify H⁺ retention (60% elevated proton transport barrier to 0.104 eV) via imposing charge‐enhanced dehydration barriers and nitrogen‐groups synergism, leveraging the higher ionic potential of protons to discriminate the conduction ions (K^+^). Simultaneously, the surface charges electrostatically guide the uniform distribution of near‐electrode zinc ions for zinc‐oriented growth without dendrites. The synergistic strategy achieves a near‐neutral zinc‐manganese flow system with a record accumulated capacity of 6510 mAh cm^−2^ (>200 cycles) at 30 mA cm^−2^, high areal capacity of 100 mAh cm^−2^ (130.1 mWh cm^−2^) at 20 mA cm^−2^, representing one of the most stable zinc‐manganese flow batteries reported. This study provides an effective membrane design strategy for low‐cost and high‐energy‐density zinc‐based flow batteries.

## Graphical abstract

An ion‐regulating membrane is designed with a surface‐concentrated positively‐charged Zn^2^⁺ crosslinked network for zinc‐manganese flow batteries. The membrane effectively mitigates proton crossover and regulates zinc ion distribution to avoid dendrites, thus significantly improving areal capacity and prolonging the lifespan with a record accumulated capacity.

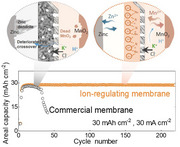

## 1 Introduction

**Figure 1:**
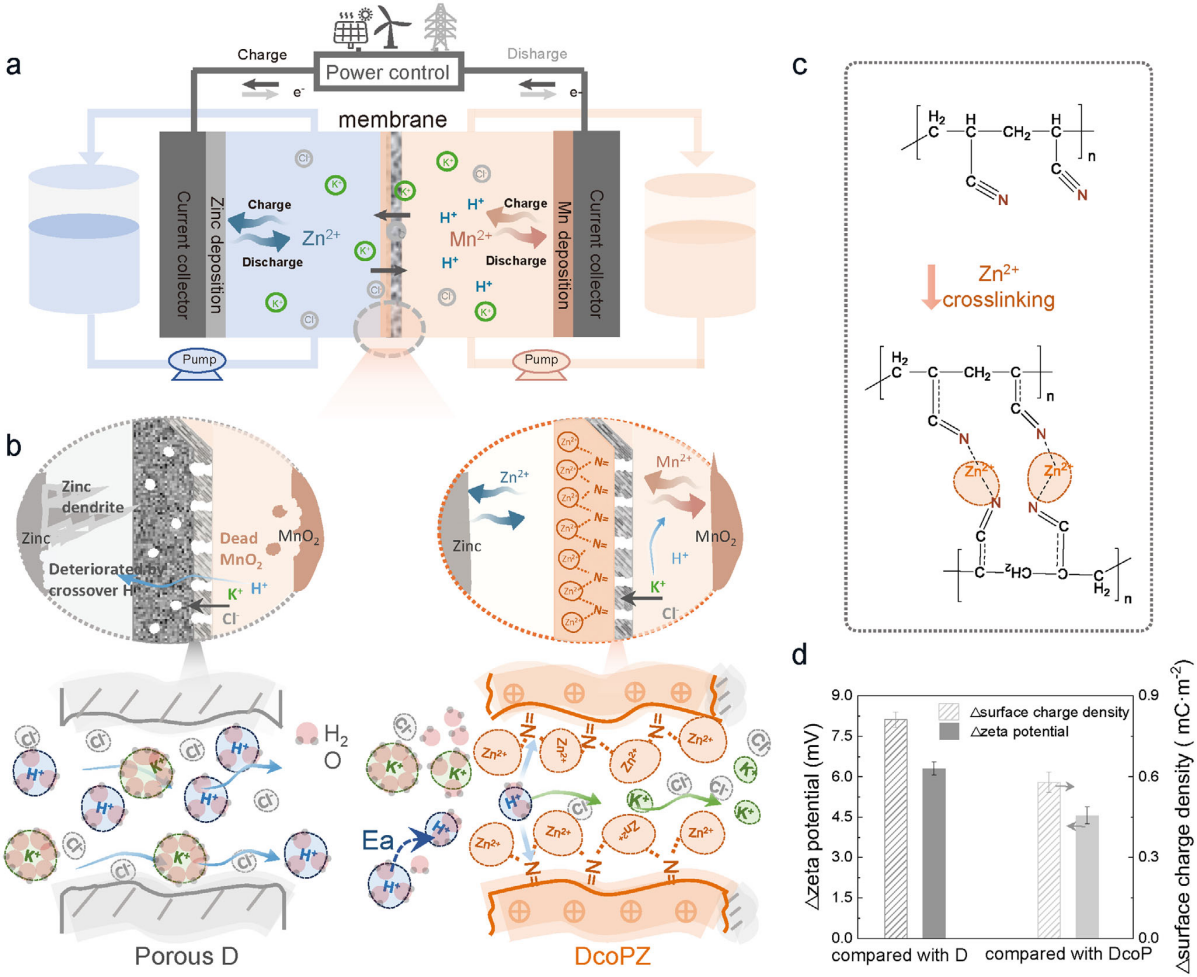
The ion‐regulating membranes with surface‐enriched positive charge designed for zinc‐manganese flow batteries. a) Illustration working principle of zinc‐manganese flow batteries. b) Schematic illustration of ion‐selective channels of membranes without (porous substrate, D) and with crosslinking network (DcoPZ), and their effects on negative zinc deposition and positive conversion of MnO_2_/Mn^2+^. All ions in electrolytes can freely cross porous polyolefin substrates (porous D). However, protons with a larger ionic potential would be decelerated by the enriched positive‐charges crosslinking network of DcoPZ, resulting from a larger transmembrane activation energy (*E*_a_) to enter the membrane, coupled with intensified interactions between protons and N‐functional groups in the crosslinked domain, which collectively amplify proton retention. c) Schematic illustration of the formation of the crosslinking network. Zn^2+^ ions are coordinated with the nitrile group (C≡N) of polyacrylonitrile (PAN) to form the crosslinked network. d) The increased zeta potential and surface charge density of DcoPZ featuring surface‐enriched positive charges, compared to that of D and uncrosslinked DcoP.

Integration of renewable energy requires the deployment of sustainable energy storage technologies owing to their intermittency.^[^ ^1^, ^2^ ^]^ Flow batteries (FBs), one of the most promising energy storage systems in meeting sustainability goals, are known for their reliability, flexibility, and rapid response to absorb energy from fluctuating renewable sources. Increasing the energy density of these technologies allows greater energy storage per unit volume, supporting longer storage durations (over 4 h) and reducing the costs of energy storage.^[^ ^3^ ^]^ Different from traditional liquid‐liquid conversion flow batteries (e.g., vanadium flow batteries with an energy density of 30–40 Wh L^−1^),^[^ ^4^ ^]^ zinc‐based flow batteries (ZFBs) effectively improve energy density by bi‐electronic transfer with the solid‐liquid conversion of earth‐abundant zinc,^[^ ^5^ ^]^ which provides a high theoretical capacity of 820 mAh g^−1^. Manganese‐based redox couples based on Mn^2+^/MnO_2_ redox (+1.23 V vs SHE) are a promising posolyte to couple with zinc negative electrode (−0.76 V vs SHE), because of its high energy density (616 mAh g^−1^) and low cost (less than $10 kWh^−1^) (**Figure** **1**a).^[^ ^6^, ^7^ ^]^

However, zinc‐manganese flow batteries (ZMFBs) face challenges of poor areal capacity of less than 20 mAh cm^−2^ (25 mWh cm^−2^) and limited lifespan (<100 cycles with accumulated capacity less than 2000 mAh cm^−2^). owing to problems of proton crossover in posolytes and zinc dendrites in negolytes. On the one hand, in posolytes, it generates H^+^ ions during Mn^2+^ deposition, which could crossover to corrode zinc electrodes and worsen the reversibility of manganese.^[^ ^8^, ^9^ ^]^ Although the strong acid‐supporting posolytes (e.g., sulfuric acid) were reported to provide sufficient protons.^[^ ^10^ ^]^ It does not fundamentally decrease proton crossover, while accelerating proton migration under a high concentration gradient, further exacerbating the stability of batteries. On the other hand, in negolytes, the uneven Zn deposition on the electrode triggers a self‐amplified surface‐roughening process, rendering by‐products (such as ZnO) and further zinc dendrites.^[^ ^8^, ^9^, ^11^, ^12^, ^13^, ^14^, ^15^, ^16^, ^17^, ^18^ ^]^ Such dendrites can potentially pierce membranes, causing short circuits in the batteries.^[^ ^19^, ^20^ ^]^ To extend the battery lifespan, researchers often supplement extra Zn foils when employing Zn salt electrolytes as the active materials,^[^ ^4^, ^11^, ^14^ ^]^ but this adds additional costs.

Herein, we designed an ion‐regulating membrane containing surface‐enriched positive charges of crosslinked networks (DcoPZ) to simultaneously mitigate the proton crossover and zinc dendrite growth (Figure 1b,c). This functional crosslinked network enables ion transfer through the membrane while discriminating protons from the conduction ions (K^+^). For protons with the larger ionic potential, surface‐enriched positive charges on membranes markedly hinder H^+^ ions dehydration, and coupled with intensified interactions between protons and N‐functional groups in the crosslinked domain, collectively amplify proton retention. Hence, it enhances the energy barrier for proton migration, showing an approximately twofold increase in the transmembrane activation energy (*E*_a_) of protons, from 0.065 to 0.104 eV. Simultaneously, the enriched positive charges on DcoPZ, leveraging the Donnan effect, establish electrostatic guidance that concentrates zinc ions toward the electrode‐adjacent interfacial zone. The effect enhances the uniform distribution of near‐electrode ions and decreases the possible concentration polarization, promoting a preferential deposition of zinc along the (0 0 2) crystal plane.^[^ ^21^ ^]^ This plane promotes dense packing, facilitating smooth zinc deposition and suppressing dendrite formation. The synergistic strategy, enabled by the membrane, achieved a near‐neutral and stable flow battery system with long‐term cycling for an accumulated charge capacity of 6510 mAh cm^−2^ over 200 cycles at a current density of 30 mA cm^−2^ and an areal capacity of 30 mAh cm^−2^. Benefiting from the synergistic strategy, as well as the merits of low areal resistance and enhanced electrolyte affinity, DcoPZ provides favorable conditions for ZMFBs to work at a high current density amounting to 100 mA cm^−2^ at 30 mAh cm^−2^. Furthermore, by altering the operational mode to simulate fluctuating renewable sources, the flow batteries show a high charge capacity of 90 mAh cm^−2^ (111.7 mWh cm^−2^) over 420 h, with plating for nearly 8 h cycle^−1^. A higher charge capacity of 100 mAh cm^−2^ (130.1 mWh cm^−2^) was achieved for nearly two weeks with a plating time of 6.4 h, representing one of the best stabilities reported for zinc‐manganese flow batteries.^[^ ^11^, ^14^, ^17^, ^22^, ^23^, ^24^, ^25^, ^26^, ^27^, ^28^, ^29^ ^]^ The DcoPZ‐enabled near‐neutral battery systems demonstrate the environmental friendliness, a compelling advantage for sustainable energy storage systems as well. More than that, the DcoPZ achieves a significant cost reduction of 77% while maintaining competitive performance, compared to conventional Nafion membranes which failed at 50 mAh cm^−2^ after piercing by zinc dendrites. On the whole, our study provides a promising strategy for sustainable energy storage by developing cost‐effective membranes for high‐energy‐density and low‐cost zinc‐based flow batteries.

## 2 Design Principles and Properties of Membranes

We propose a membrane design strategy, constructing surface‐enriched positive charges of crosslinked networks (DcoPZ, Figure 1b), to mitigate proton crossover in posolytes and zinc dendrites in negolytes. In the strategy, a low‐cost and robust porous substrate was employed as the substrate (D) for ion transport and to resist possible zinc dendrites. The positively charged surface layer was designed to exert a stronger retention effect on cations with a larger ionic potential (*Z*/*r* ^2^, ionic charge *Z* and ionic radius *r*), i.e., H^+^ has a larger ionic potential (8.70 nm^−1^) than K^+^ ions (6.71 nm^−1^) (Table S1, Supporting Information).^[^ ^30^ ^]^ For cations with higher ionic potential, the enriched positive charge on the membrane significantly impedes ion dehydration to enter the membrane, while the nitrogen‐containing groups within the crosslinked domain interact with and amplify the proton retention effect. Thus, it allows ion transfer through the membrane while discriminating proton (H^+^) from the conduction ions (K^+^). In addition, the surficial localized positive charges induce zinc‐oriented growth without dendrites, by electrostatically guiding that concentrates the zinc ions toward the electrode‐adjacent interfacial zone, which effectively uniform distribution of near‐electrode ions and decreases the concentration polarization. The synergistic strategy has proved effective in facilitating the performance of the ZMFBs.

To construct the membrane, the crosslinking between polyacrylonitrile and Zn^2+^ ions was applied to form a thin layer to provide the surficial localized positive charges on the polyolefin substrates (Figure 1c). Notably, due to the strictly required coordination conditions, the membrane fabrication parameters, especially the organic solvent and reaction time, are identified as critical factors in the generation of the crosslinking network (Table S2 and Figures S2–S5, Supporting Information, for further details and DFT calculation). The formation of continuous Zn^2+^‐crosslinked networks on the DcoPZ endowed the membranes with surficial localized positive charges. For better descriptions, uncrosslinked DcoP membranes with a top layer of polyacrylonitrile on the porous substrate (D) were prepared as control samples. The zeta potential of DcoPZ increased by ≈6.3 mV, together with an enhanced surface charge density of around 0.81 mC m^−2^ compared to D (Figure 1d). Whereas D and DcoP exhibited minimal differences in zeta potential and surface charge density. As we anticipate, the crosslinking plays a critical role in shaping the surficial localized positive charges of membranes.

**Figure 2:**
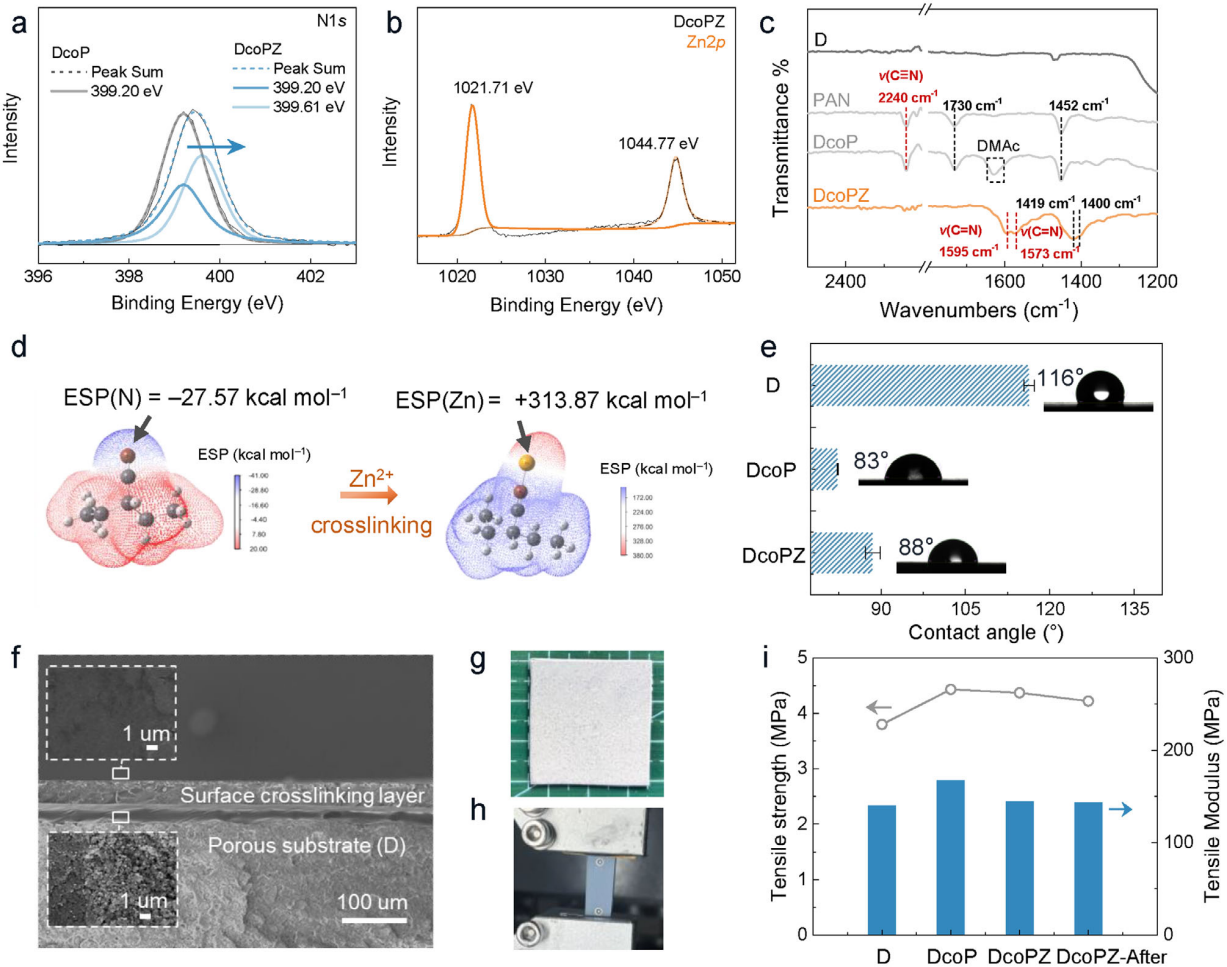
The formation mechanism and properties of ion‐regulating membranes with surface‐enriched positive charge. a) The N 1s spectra of XPS on DcoP and DcoPZ. b) The Zn 2p spectra of XPS on DcoPZ. c) The FTIR spectra of the membrane surface. The peak of C≡N disappeared and the peak of C═N was detected after crosslinking. d) The surface electrostatic potential of the simplified PAN unit and PAN unit‐Zn^2+^. e) The contact angle of membranes tested in 2 m KCl. Error bars, mean ± standard deviation (s.d.). f) The scanning electron microscopy (SEM) images for the cross‐section morphology of DcoPZ. The insets are the enlargement of the position being defined, including the porous substrate and the dense surface layer. g) Photos of DcoPZ membrane. Characterization of the mechanical properties of membranes. h) Photos during the tests. i) The tensile strength and tensile modulus of membranes.

The membrane formation mechanism was studied with X‐ray photoelectron spectroscopy (XPS) (**Figure** **2**a,b) and Fourier transform infrared (FTIR) spectroscopy (Figure 2c), confirming the binding of Zn^2+^ ions to the nitrile groups of PAN. According to the XPS spectra of DcoPZ (Figure 2a,b), the binding energy of the N 1s peak increased from 399.20 eV of DcoP to 399.61 eV, implying that the electrons of nitrogen atoms occupied the empty orbitals of Zn^2+^, causing a decrease in electron density around nitrogen atoms. The coordination of Zn^2+^ with PAN on the membrane is stable, which was proved by the identical Zn 2p peaks before and after battery test (Figure 2b; Figure S6, Supporting Information). The results of the FTIR spectroscopy further supported the conclusion of the XPS. In the FTIR spectrum of DcoP, a peak around 2240 cm^−1^ was detected, which was assigned to the nitrile group of the PAN (Figure 2c).^[^ ^31^, ^32^, ^33^ ^]^ After the coordination of Zn^2+^ with PAN for DcoPZ, it resulted in the disappearance of the C≡N peak and the emergence of C═N peaks at 1595 and 1573 cm^−1^, verifying that Zn^2+^ coordinated with C≡N of PAN.^[^ ^32^ ^]^ The double peaks of C═N indicated that two identical C═N were connected to the Zn^2+^ ions, producing strong stretching vibration coupling and splitting the vibration absorption peak.^[^ ^34^ ^]^ Subsequently, density functional theory (DFT) calculations were conducted to provide further insights into the reaction. The results indicated that the nitrogen atoms of nitrile groups (C≡N) exhibited an average electrostatic potential of −27.57 kcal mol^−1^, which facilitated the binding of Zn^2+^ ions with them, showing the binding energy of −370.99 kcal mol^−1^. In the final structure of crosslinking, the average electrostatic potential around the Zn atom is positive (Figure 2d), which plays a vital role in fabricating surface‐enhanced positive charges on membranes.

After construction of the surface functional layer, the DcoPZ exhibited a significantly smaller contact angle than that of the pristine D membrane, indicating that the surface functional layer improves the membrane hydrophilicity, which helps to reduce the resistance in batteries (Figure 2e). The slightly higher contact angle of DcoPZ (88°) compared to DcoP (83°), which may be related to the formation of a crosslinked structure. The morphology of the designed DcoPZ membrane was further observed via scanning electron microscopy (SEM) (Figure 2f; Figure S7, Supporting Information). Compared to the pristine D, the DcoPZ exhibited an even and dense crosslinking layer with a thickness of ≈35 µm. Inductively coupled plasma mass spectrometry (ICP‐MS) results verified that the Zn content in the crosslinking layer was about 0.142%. In addition, membranes maintained excellent thermal stability comparable to the pristine D, according to the thermal gravimetric analysis (TGA) (Figure S8, Supporting Information). The mechanical properties were further evaluated by the Universal Testing Machine (Figure 2g,h). Due to the low polymer mass fraction (2 wt%) and the thin functional layer (35 µm), the tensile strength is primarily governed by the robust substrates used in membrane fabrication. Figure 2i revealed that the DcoPZ maintains superior mechanical tensile strength and comparable tensile modulus to resist possible zinc dendrites for the long‐term operation of the battery.

## 3 Alleviating the Proton Crossover

Previous studies have revealed that the protons exert a significant influence on the performance of ZMFBs, particularly regarding zinc corrosion and manganese reversibility.^[^ ^35^, ^36^ ^]^ During the plating process, H^+^ ions are produced as Mn^2+^ is oxidized to MnO_2_. These H^+^ ions easily cross the membrane and enter the negolyte, where they corrode the deposited zinc and lead to the capacity loss of batteries. Moreover, the loss of H^+^ ions in the posolyte causes insufficient H^+^ ions to promote the reduction of MnO_2_ to Mn^2+^ during battery discharge. In this circumstance, poor reversibility of MnO_2_/Mn^2+^ reaction triggers a cascade of failure mechanisms such as the formation of dead MnO_2_, which limits the battery performance, particularly under harsh conditions of high charge areal capacity and high current density. Therefore, it is crucial to mitigate proton crossover in the posolyte.

**Figure 3:**
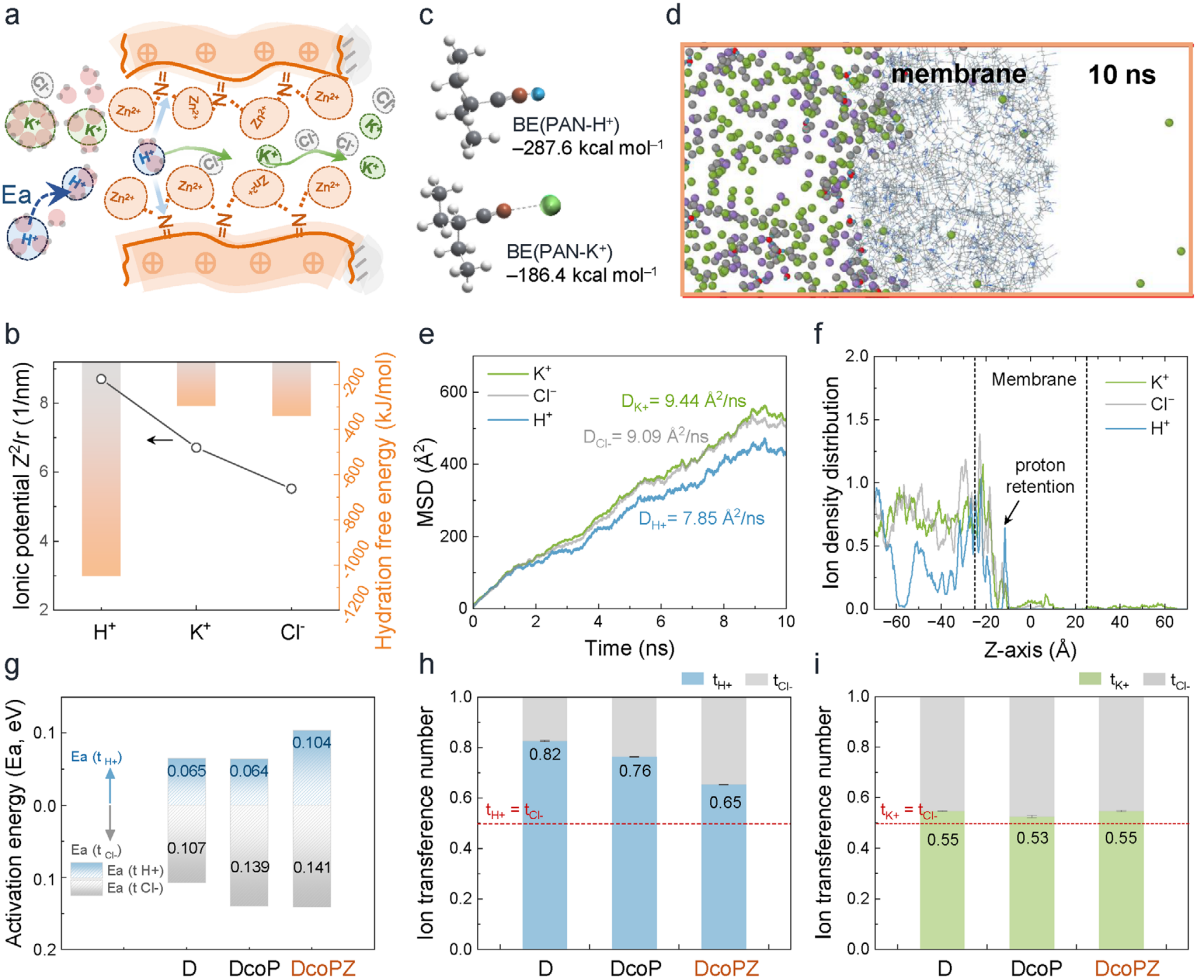
Effects of surface‐enriched positive charges of membranes on ion transport behaviors. a) Schematic illustration of ion selective channels of the surface layer with the enriched positive charges in DcoPZ membranes. b) Comparison of ionic potential (Z^2^/r)^[^ ^37^, ^40^ ^]^ and the hydration free energy^[^ ^39^ ^]^ of H^+^, K^+^, and Cl^−^ ions. c) The binding energy of H^+^ and K^+^ ions with PAN. d) A molecular dynamics (MD) simulation snapshot showing ion diffusion across the DcoPZ membrane at 10 ns. The system was constructed with an electrolyte solution on the right side of the membrane and pure water on the left. All ionic and molecular species other than H^+^, K^+^, and Cl^−^ ions are hidden. e) The mean square displacement (MSD) of ions and f) the ion density distribution obtained from the simulation statistical results. g) Comparison of the transmembrane activation energy of H^+^ and Cl^−^ ions across D, DcoP, and DcoPZ membranes. h) The H^+^ (*t*_+_) and Cl^−^ (*t*_−_) ion transference number and i) K^+^ (*t*_+_) and Cl^−^ (*t*_−_) ion transference number for D, DcoP, and DcoPZ membranes. Measurements were tested in the HCl and KCl solutions, respectively, with a gradient of 0.01 and 0.1 mol L^−1^.

The designed DcoPZ membrane with a surface layer of enriched positive charges helps to reduce proton crossover without affecting the conduction of charge carriers potassium ions. The porous polyolefin substrate allows hydrated ions to freely transport through, while the surface‐enhanced charge networks exhibit a retention effect on the hydrated ions with a larger ionic potential (*Z* ^2^/*r*), especially for H^+^ ions (**Figure** **3**b).^[^ ^37^, ^38^ ^]^ The positively charged surface significantly imposes dehydration resistance for the proton entry membrane and further enhances proton retention within the membrane by intensified interactions between ions and nitrogen‐containing groups in the crosslinked domain.

For ion transmembrane transport behavior, it is generally recognized that hydrated ions need to dehydrate, then enter and traverse the membrane. Especially considering the hydrated ions aggregate near the membrane in the solution concentration greater than 0.05 m, easily resulting in a retention effect of ions across the membrane.^[^ ^37^, ^38^ ^]^ On the one hand, the H^+^ ions, which feature a larger ionic potential of 8.70 nm^−1^ than charge carriers K^+^ ions of 6.71 nm^−1^, would experience stronger electrostatic repulsion by the positively charged surface layer. Moreover, the larger ionic potential of H^+^ ions endows them with stronger ion–solvent interactions, exhibiting a more negative hydration energy (−1063.0 kJ mol^−1^) compared to that of K^+^ ions (−307.5 kJ mol^−1^) (Figure 3b; Table S1, Supporting Information).^[^ ^39^ ^]^ That means, the protons encounter more powerful electrostatic repulsion by surficial localized positive charges, together with a greater difficulty in dehydration for transmembrane. On the other hand, when ions migrate into the membrane, the nitrogen atoms in the polymer network interact with K^+^ and H^+^ ions, further affecting the transport of cations within the membrane. The results showed that since the ionic potential energy of H^+^ ions is greater than that of K^+^ ions, the N‐binding energy of H^+^ ions (−287.6 kcal mol^−1^) was significantly stronger than that of K^+^ ions (−186.4 kcal mol^−1^) (Figure 3c), which would further retard the transmembrane process of protons. In conclusion, leveraging the larger ionic potential of protons, the surface‐enriched positive charges on the membrane not only markedly impede proton dehydration to enter the membrane but also exhibit additional retention arising from stronger interactions for protons with nitrogen atoms within the crosslinked domain. The dual collaborative mechanisms facilitate a pronounced retention for proton transmembrane migration, while ensuring K^+^ ions permeation completes the internal current circuit. Similarly, the charge carriers Cl^−^ ions with the lowest ionic potential of 5.52 nm^−1^, showed a low hydration energy of −352.7 kJ mol^−1^, allowing them to permeate membranes effortlessly, alongside K^+^ ions.

The retention effect was evidenced by the H^+^ ions permeation test (Figure S9, Supporting Information). The diffusion rate of protons traversing the DcoPZ was 1.269 ± 0.1657 mol m^−2^ h^−1^, which is decreased by half compared to that of pristine D (2.553 ± 0.0423 mol m^−2^ h^−1^) and DcoP (2.276 ± 0.0375 mol m^−2^ h^−1^). To gain molecular‐level insights into the ion transport behavior, molecular dynamics (MD) simulations were performed to investigate ion diffusion through the DcoPZ membrane (Figure 3d). The mean squared displacement (MSD) of ions was analyzed to determine their diffusion coefficients (Figure 3e). The results revealed that the charge carriers, K⁺ and Cl^−^, exhibited the highest and comparable diffusion coefficients (9.44 Å^2^ ns^−1^ for K⁺ and 9.09 Å^2^ ns^−1^ for Cl^−^). In comparison, the proton diffusion coefficient was 16.8% lower than that of K⁺ (7.85 Å^2^ ns^−1^). These simulation results are consistent with our experimental observations. Furthermore, the spatial distribution of ions after 10 ns of diffusion was analyzed. As shown in Figure 3f, a pronounced retention effect is evident for protons at locations indicated by the arrows, which can be reasonably attributed to interactions between protons and nitrogen atoms within the membrane.

To gain insight into the ion transport behavior of membranes and to elucidate the retention mechanism of H^+^ ions, the transmembrane activation energy (*E*_a_) and ion transference number were further studied. As illustrated in Figure 3g, DcoP presented a similar proton transport barrier while increasing the Cl^−^ transport barrier compared to that of pristine D, because of a PAN layer without crosslinked networks. That means a general surface layer without surface‐enhanced positive charges would not function in mitigating proton crossover. However, compared to DcoP, the *E*_a_ of H^+^ ions for DcoPZ increased from 0.064 to 0.104 eV with the assistance of Zn^2+^‐crosslinked networks, representing a nearly twofold increase, whereas the Ea of Cl^−^ ions remained unchanged. These findings verified that the attachment of zinc ions to PAN raised the difficulty of proton transport without significantly impairing the Cl^−^ ions transport.

More results revealed that the charge carriers K^+^ ions could still efficiently traverse DcoPZ when the transmembrane diffusion of protons is restricted. The ion transference numbers of membranes in HCl and KCl solutions were further investigated (Figure 3h,i). The utilization of DcoPZ, as opposed to D, resulted in a decrease of the ion transference number of H^+^ ions (*t*_H+_) from 0.82 to 0.65 in HCl solution. It is noteworthy that this alteration did not influence the transport characteristics of K^+^ ions, according to the consistent ion transference number of K^+^ ions (*t*_K+_) in the KCl solution. A comparable trend was observed when comparing DcoPZ to DcoP. In addition, the DcoPZ exhibited a great affinity with KCl supporting electrolytes, according to a smaller contact angle and lower areal resistance of DcoPZ in comparison to D in a 2 mol L^−1^ KCl solution (Figure 2e; Figure S10, Supporting Information). Although DcoPZ exhibits a slightly higher contact angle (88°) than DcoP (83°), its crosslinked network appears to provide more efficient ion transport pathways for KCl, leading to higher ionic conductivity. These findings suggested that the surficial positive charges of DcoPZ successfully diminished the transmembrane diffusion of H^+^ ions without compromising charge carriers K^+^ ions conduction.

Overall, with the help of designed enriched surficial positive charges, the protons display a tendency to be retained when passing through DcoPZ, thereby increasing the barrier to transmembrane transport. In contrast, the charge carriers K^+^ ions (alongside Cl^−^ ions), which are less affected by the ion‐water interactions for entering membranes and the ion‐nitrogen atoms interactions within membranes, can readily strip off the water molecules to pass through the membranes and achieve high conductivity. Given that DcoPZ effectively alleviates proton transmembrane diffusion, a visualization experiment and UV–vis spectra were further conducted to show the impact of DcoPZ on the reversible conversion of manganese (see Figure S11, Supporting Information, for further information). The results verified that the retention behavior of H^+^ ions on DcoPZ decreased the generation of dead MnO_2_, which is expected to facilitate the manganese reaction of ZMFBs in a long‐term operation.

## 4 Mitigating Zinc Dendrites

Previous zinc dendrite formation persists as a critical failure mode for zinc‐based batteries, especially at high areal capacities.^[^ ^41^ ^]^ At the membrane–electrode interface, localized deposition induces irregular dendrites due to uneven distribution of Zn^2+^ flux and current density, readily piercing membranes and harming battery stability.^[^ ^42^ ^]^ Beyond suppressing proton crossover in posolytes to enhance zinc corrosion resistance and Mn reversibility, DcoPZ enables oriented zinc deposition through interfacial distribution uniformity. This dual‐functionality facilitates batteries operating at higher charge areal capacities and effectively enhances their energy density.

**Figure 4:**
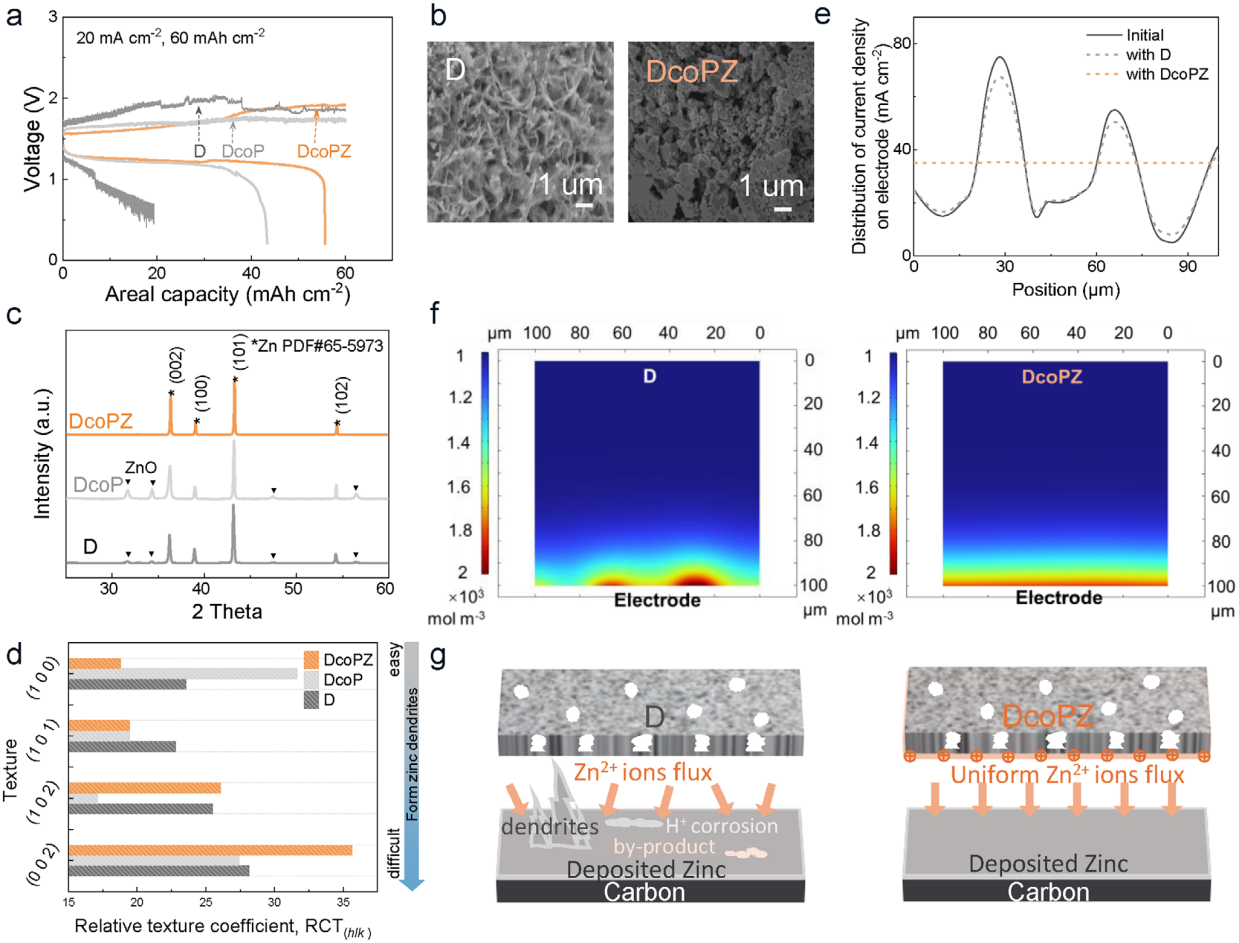
Effects of membranes with surface‐enriched positive charges of membranes on zinc deposition. a) Voltage profiles of zinc‐manganese flow batteries (ZMFBs) assembled with D, DcoP, and DcoPZ membranes (20 mA cm^−2^, 60 mAh cm^−2^). b) The morphology of zinc deposited on the carbon felt was examined using SEM following the charging phase of the 15th cycle (20 mA cm^−2^, 60 mAh cm^−2^). c) XRD spectra of deposited zinc. d) The relative texture coefficient (RCT) alone Zn (1 0 0), Zn (1 0 1), Zn (1 0 2), and Zn (0 0 2) has been calculated according to (b). The inset is the schematic illustration of the hexagonal close‐packed (h c p) structure of metal Zn. e) Simulation of the current density distribution on the electrode at the initial (solid) and after a time affected with D or DcoPZ (short dash). f) Simulation of the concentration distribution of zinc ions at the membrane–electrode interface for the DcoPZ and D when the battery charges. g) Schematic diagram of the zinc deposition process affected by membranes.

To demonstrate the superiority of DcoPZ, the zinc‐zinc symmetric flow batteries were assembled with different membranes (Figure S12, Supporting Information). The results showed that the DcoPZ membrane demonstrates lower polarization and superior cycling stability compared to D and DcoP membranes. Furthermore, the ZMFBs were assembled with D, DcoP, and DcoPZ, operating at a high charge areal capacity of 60 mAh cm^−2^. Under this condition, the D and DcoP were pierced by zinc dendrites, causing short circuits in batteries. However, DcoPZ allowed the battery to work stably without fluctuations in the voltage profile (**Figure** **4**a). The morphology of deposited zinc was observed by SEM and illustrated in Figure 4b and Figure S13 (Supporting Information). When the positively charged surface layer of DcoPZ was positioned facing the anode, the zinc deposited compactly and evenly, compared to the loosened and protuberant deposits observed with D and DcoP. The distinct zinc morphology suggested a difference in zinc crystallinity. The zinc crystalline structure was further analyzed by X‐ray diffraction (XRD) to investigate the differences in zinc deposition modulated by membranes. The results presented in Figure 4c implied that only the diffraction peaks of metallic zinc were detected when employing DcoPZ, with almost no generation of ZnO by‐products. However, obvious diffraction peaks of ZnO at 30°–35° were detected for D and DcoP, suggesting that unexpected side reactions occurred at the membrane–electrode interface, such as hydrogen evolution reaction (HER).^[^ ^43^ ^]^ Subsequently, the relative texture coefficient (RCT) was calculated for the planes of Zn (1 0 0), Zn (1 0 1), Zn (1 0 2), and Zn (0 0 2) based on the XRD data.^[^ ^44^ ^]^ Figure 4d displayed the preferred orientation of Zn (0 0 2) plane regulated by DcoPZ. It is speculated that when DcoPZ faced the negative side, the surface‐enriched positive charges facilitated the uniform distribution of interfacial Zn^2+^ ions via electrostatic interaction, analogous to that observed in other studies.^[^ ^5^, ^45^ ^]^ This regulatory effect drove the zinc atoms to arrange in a configuration oriented with the Zn (0 0 2) plane, which was a compact packing arrangement. The atomic packing endowed the zinc deposits with a relatively smooth surface and uniform interfacial charge density, further conducive to subsequent even zinc deposition without the generation of zinc dendrites (Figure 4d).^[^ ^46^, ^47^ ^]^ Nevertheless, in the absence of the regulatory effect, both D and DcoP caused zinc to grow with preferential orientation along the (1 0 0) plane. The Zn (1 0 0) plane features uneven interfacial charge density and has been reported to render nonuniform interfacial Zn^2+^ ions.^[^ ^46^, ^47^ ^]^ It causes the membranes to be susceptible to zinc dendrite growth, which deteriorates battery operation.

The regulatory effect of membranes governing zinc ion deposition was further elucidated. Generally, during battery operation, zinc ions predominantly undergo electrochemical reactions at the electrode, while abundant smaller potassium ions and chloride ions serve as charge carriers. For one reason, due to the large size of zinc ions, their transmembrane permeation rate in the permeation test is significantly slow (Table S3, Supporting Information). For another reason, considering the symmetrical electrolyte in ZMFBs, the transmembrane transport of zinc ions driven by concentration gradients is less. Therefore, when battery charging, the Zn^2+^ ions migrate towards the electrode in negolyte, where they receive electrons and deposit on the electrodes under the action of the internal electric field from the positive to the negative side. Beyond the internal electric field, membranes regulate the distribution of zinc ions as well. Given that the ion permeation rate (*J*) of zinc ions traversing DcoPZ decreased by one‐third (0.182 ± 0.013 mol m^−2^ h^−1^, Table S3, Supporting Information) compared to that of D in the permeation test, this indicates that the positive charges on the membrane did influence the diffusion and distribution of zinc ions. According to the Donnan effect, the charge distribution across a membrane would affect the distribution of nearby ions. Hence, the evenly distributed positive charges on DcoPZ establish electrostatic guidance that concentrates zinc ion flux toward the electrode interface. This effect promotes near‐electrode ion uniform distribution and decreases the possible concentration polarization, enabling their uniform electrodeposition.

To elucidate interfacial ion modulation mechanisms, a two‐dimensional model was implemented to simulate the dynamic distribution of current density and zinc ions at the membrane–electrode interface via the finite‐element method. The recombination electric field, originating from the membrane surface charges and internal electric field coupling, is reflected in the electrode reaction parameters, governing zinc ions/current density distribution evolution. Figure 4e exhibits that the initial non‐uniform current density distribution evolves under DcoPZ membrane regulation toward spatial uniformity, whereas the D membrane sustains non‐uniformity. This divergence stems from fundamental differences in the distribution and consumption of zinc ions at the membrane–electrode interface. As shown in Figure 4f, the positive‐charge‐enhanced interface on DcoPZ regulates the consistency of zinc ions within an ultrathin 2 µm electrode‐adjacent layer, facilitating uniformity for zinc ion consumption and consequent current density distribution. This cascade explains the compact and dendrite‐free zinc deposition observed in batteries assembled with DcoPZ. Conversely, D membrane permits zinc ions to disperse across ≈10 µm zones, intensifying concentration gradients during charging. The significant nonuniform distribution of zinc ions causes an uneven distribution of the current density. It induces localized deposition, finally triggering irregular dendrite growth.

In summary, the designed DcoPZ takes advantage of surficial enriched positive charges could regulate interfacial zinc deposition. By electrostatically guiding zinc ions toward the electrode‐adjacent interfacial zone, it induces uniform distribution and consumption of zinc ions and zinc‐oriented growth (Figure 4g). In terms of long‐term operation, DcoPZ, with the ability to effectively suppress dendrite formation, shows promising potential in realizing stable battery performance at high areal capacities.

## 5 Demonstration of High‐Energy‐Density Zinc‐Manganese Flow Batteries

**Figure 5:**
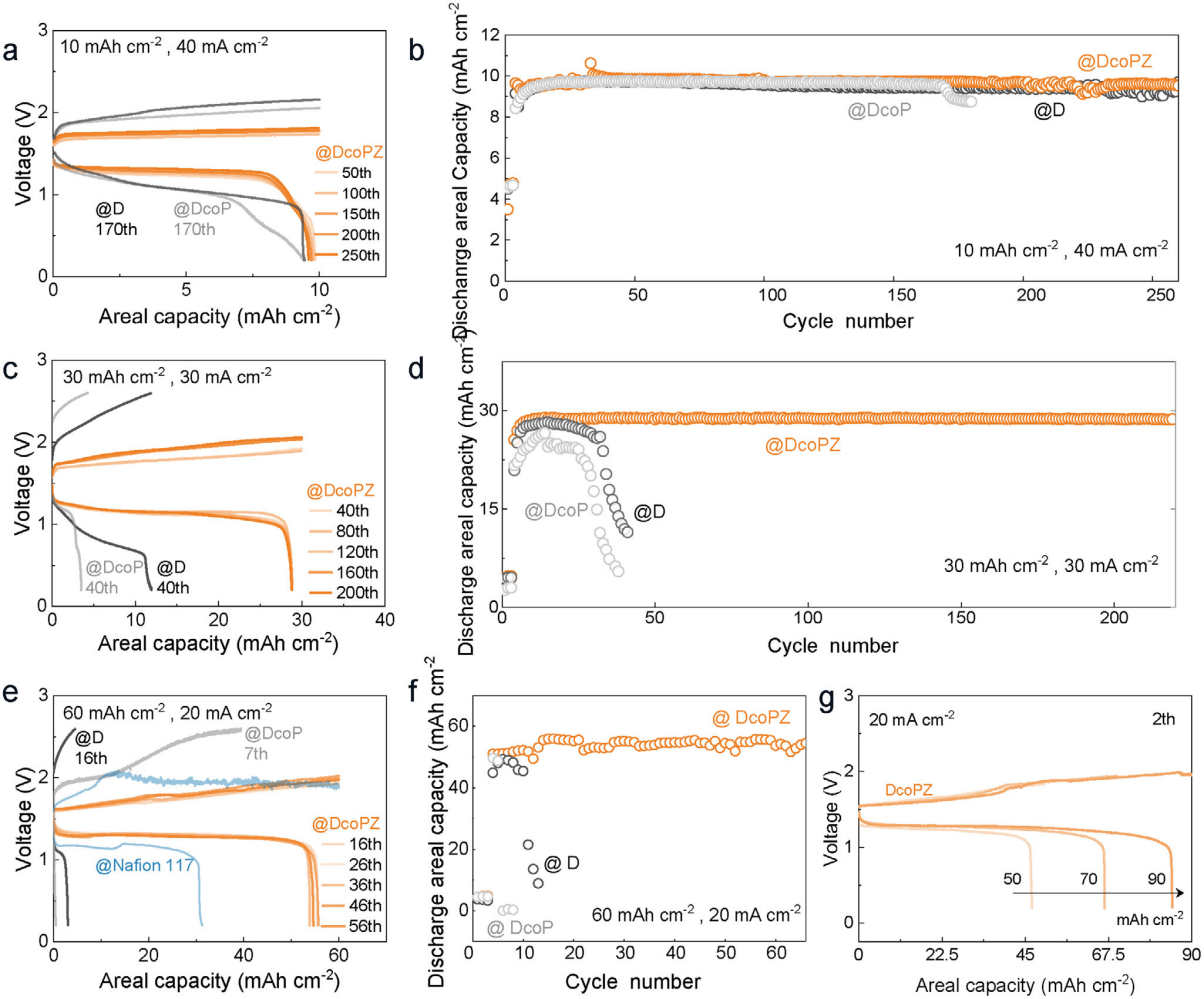
Applications of membranes for zinc‐manganese flow batteries (ZMFBs). a) Voltage profiles and b) cycling performance of ZMFBs assembled with D, DcoP, and DcoPZ membranes (40 mA cm^−2^, 10 mAh cm^−2^). c) Voltage profiles and d) cycling performance of ZMFBs assembled with D, DcoP, and DcoPZ membranes (30 mA cm^−2^, 30 mAh cm^−2^). e) Voltage profiles and f) cycling performance of ZMFBs assembled with D, DcoP, and DcoPZ membranes (20 mA cm^−2^, 60 mAh cm^−2^). g) Voltage profiles of ZMFBs assembled with DcoPZ at the high areal capacities of 50, 70, and 90 mAh cm^−2^.

Previous The designed DcoPZ membranes, featuring surface‐enriched positive charges crosslinking networks, could not only reduce the H^+^ ions crossover but also prevent dendrite formation, thus effectively improving the performance of near‐neutral zinc‐manganese flow batteries. At a relatively high current density of 40 mA cm^−2^, which involves rapid plating/stripping processes, battery performance is impacted by the transmembrane transport ability of charge carriers, even when the areal charge capacity is not exceeded (e.g., 10 mAh cm^−2^ in this case). Because DcoPZ manifests lower areal resistance and superior electrolyte affinity, the polarization of batteries using DcoPZ remains almost unchanged after 260 cycles of quick plating and stripping, contributing to the long‐term stability of batteries, as proved by the voltage profiles in **Figure** **5**a.

However, the D and DcoP hinder charge carriers in comparison to DcoPZ, resulting in significantly increased internal overpotential in batteries. The cycling data presented in Figure 5b indicated that batteries using the D exhibited a lower discharge capacity than those employing DcoPZ. As for DcoP, it performed even worse, showing a pronounced decline in discharge capacity around 160 cycles. This decline likely comes from membrane fouling within the DcoP, such as surface contamination or structural deterioration, which progressively worsens with use, causing incremental polarization and the decreased discharge platform (Figure 5a).

Elevating the areal capacity of batteries to 30 mAh cm^−2^ was accompanied by a corresponding rise in deposition amounts for zinc and MnO_2_. The distribution of zinc ions at the membrane–electrode interface became more erratic, thus exacerbating zinc dendrite formation. In addition, the H^+^ ions (≈0.89 mol L^−1^) produced alongside the MnO_2_ deposits, partially traversed the membrane to reach the negolyte, where they corroded the zinc electrode. This irreversible loss of H^+^ ions was also detrimental to the manganese reversibility, leaving nonconductive MnO_2_ on the positive electrode. As shown in Figure 5c, neither D nor DcoP could relieve the formation of zinc dendrites or the crossover of H^+^ ions, therefore, the internal polarization of batteries increased rapidly, which reached the cut‐off voltage before the target charge capacity was achieved. Nevertheless, DcoPZ exhibited superior performance at a charge capacity of 30 mAh cm^−2^. The designed membranes regulated the distribution of interfacial zinc ions without forming zinc dendrites. Moreover, since DcoPZ diminished the transmembrane diffusion of H^+^ ions over an extended period, it decreased the quantity of MnO_2_ residue, thus less irreversible capacity decay in posolyte. The internal polarization of batteries assembled with DcoPZ was markedly reduced. The DcoPZ‐based flow batteries achieved an energy efficiency of 65.67% at the 8th cycle, significantly higher than those of D (54.92%) and DcoP (49.95%). The batteries displayed consistent performance over 200 cycles with a high accumulated capacity of 6510 mAh cm^−2^ (Figure 5c,d).

As the areal charge capacity is further increased, the issue of proton crossover and zinc dendrites crucially impacts the stability of batteries, particularly dendrite formation during brief start‐up periods. The reduction of zinc ions to zinc metal on the electrode results in a quick decline in the concentration of zinc ions in the vicinity of the electrode, increasing interfacial polarization. Higher areal charge capacity deteriorates interfacial polarization, which in turn accelerates the formation of zinc dendrites. To illustrate such an issue, flow batteries were assembled with commonly employed commercial Nafion membranes. (Figure S14, Supporting Information; Figure 5e). The results suggested that the inadequate mechanical stability of Nafion leads to a battery short‐circuit at 50 mAh cm^−2^. Despite the pristine D used in this study featuring better mechanical properties than Nafion, they still failed to meet operational requirements when the areal charge capacity was increased to 60 mAh cm^−2^ (Figure 5f). More than that, the inferior affinity of D for the electrolyte exacerbated the polarization phenomenon, causing the cut‐off voltage to be reached prematurely and a reduction in discharge capacity, showing an energy efficiency of 49.25% at the 8th cycle. Similarly, DcoP was also susceptible to overwhelming zinc dendrites and failed after seven cycles. In contrast, the batteries assembled with DcoPZ showed a high degree of operational stability, maintaining a charge capacity of 60 mAh cm^−2^ for 66 cycles (365 h), providing a higher energy efficiency of 63.92% at the same cycle. Significantly, DcoPZ enabled the ZMFBs to function when the charge capacity was even elevated to 70 and 90 mAh cm^−2^ at a current density of 20 mA cm^−2^, which represents one of the highest stabilities reported in recent years (Figure 5g).^[^ ^8^, ^9^, ^11^, ^12^, ^14^, ^15^, ^16^, ^17^, ^18^, ^26^, ^27^, ^28^, ^29^ ^]^

**Figure 6:**
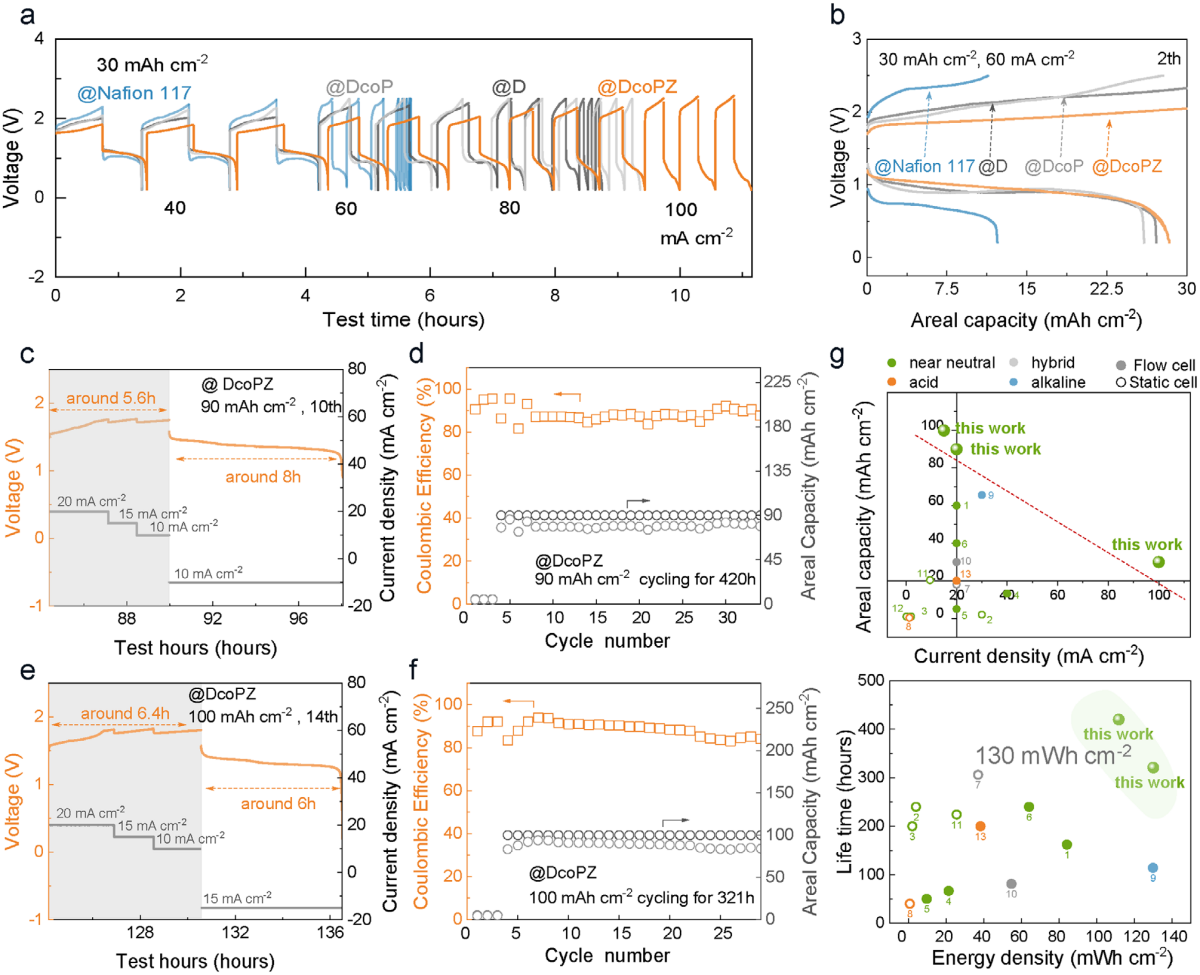
Demonstrations of high‐energy‐density and low‐cost zinc‐manganese flow batteries (ZMFBs) for sustainable energy storage. a) Voltage profiles of ZMFBs at the various current densities assembled with Nation 117, D, DcoP, and DcoPZ membranes. b) Comparison polarization of ZMFBs at 60 mA cm^−2^ with different membranes. Voltage profiles of ZMFBs assembled with DcoPZ at the high charge capacity of c) 90 mAh cm^−2^ and d) the cycling performance for 420 h (≈17.5 d). Voltage profiles of ZMFBs assembled with DcoPZ membranes at the high charge capacity of e) 100 mAh cm^−2^ and f) the cycling performance for 321 h (≈13.4 d). g) Performance comparison of areal capacity and current density for ZMFBs reported recently. Performance comparison of lifetime and energy density for ZMFBs reported recently. References: 1,^[^ ^11^ ^]^ 2,^[^ ^12^ ^]^ 3,^[^ ^26^ ^]^ 4,^[^ ^14^ ^]^ 5,^[^ ^15^ ^]^ 6,^[^ ^16^ ^]^ 7,^[^ ^8^ ^]^ 8,^[^ ^9^ ^]^ 9,^[^ ^17^ ^]^ 10,^[^ ^18^ ^]^ 11,^[^ ^28^ ^]^ 12,^[^ ^27^ ^]^ 13.^[^ ^29^ ^]^

Taking advantage of surface‐enriched positive charges, together with the merits of lower areal resistance and enhanced electrolyte wettability, DcoPZ provides favorable conditions for ZMFBs to work at high current densities from 40 to 100 mA cm^−2^ at 30 mAh cm^−2^ (**Figure** **6**a). On the contrary, the batteries equipped with the contrast membranes failed to meet such rapid plating/stripping processes, and were unable to work when the current density exceeded 60 mA cm^−2^. As shown in Figure 6b, voltage profiles provide specific information on performance limitations, revealing dominant polarization differences. The batteries enabled by DcoPZ manifest a significantly lower polarization than other membranes. Therein, the DcoP and commercial Nafion 117 membranes exhibited a high membrane resistance under the condition of high current density, thereby reaching the cut‐off voltage before achieving the anticipated capacity. We further conducted a long‐term cycling test of 600 cycles to evaluate the stability of DcoPZ (Figure S15, Supporting Information). The results indicate that the overall performance remains stable throughout the cycling test.

Given the variable nature of renewable energy sources, which give rise to fluctuating charge processes for energy storage, batteries are confronted with challenges in adapting to different charge current densities. Here, the operational mode of the batteries was altered to accommodate flexible charge current densities. As shown in Figure 6c, with DcoPZ, it is feasible for batteries to plate for 5.6 h (at a flexible charge current density from 10 to 20 mA cm^−2^) and strip for nearly 8 h at 10 mA cm^−2^. This highlights an ultrahigh charge capacity of 90 mAh cm^−2^ for steady operation over 420 h (≈17.5 d), offering an energy density of 111.7 mWh cm^−2^ (Figure 6d). A longer plating time of 6.4 h for ZMFBs was further achieved (Figure 6e,f), yielding an exceptional charge capacity of 100 mAh cm^−2^. These batteries realized a discharge process of ≈6 h and an energy density of 130.1 mWh cm^−2^ in an operational period exceeding 321 h (≈13.4 d), surpassing other reported values (Figure 6g).^[^ ^8^, ^9^, ^11^, ^12^, ^14^, ^15^, ^16^, ^17^, ^18^, ^26^, ^27^, ^28^, ^29^ ^]^

To illustrate the competitive advantage of sustainable energy storage technologies realized by DcoPZ, we further comprehensively compared and evaluated the performance of ZMFBs and several other representative zinc‐based flow batteries (Table S4 and Figure S16, Supporting Information).^[^ ^11^, ^14^, ^17^, ^22^, ^23^, ^24^, ^25^, ^26^, ^27^, ^28^, ^29^ ^]^ The performance of these batteries was assessed based on five key battery performance indicators, namely areal capacity (mAh cm^−2^), current density (mA cm^−2^), cycle life (hours), accumulate charge capacity (mAh cm^−2^), energy density (mWh cm^−2^), and two cost indicators including electrolyte cost ($ kWh**^−^** ^1^) and membrane cost ($ m**^−^** ^2^). Four types of zinc‐based flow batteries were considered: zinc‐manganese flow batteries (Zn‐Mn) in alkaline, acid, or neutral medium, zinc‐bromine flow batteries (Zn‐Br), zinc‐iodine flow batteries (Zn‐I), zinc‐iron flow batteries (Zn‐Fe) in alkaline or neutral medium. Compared to reported ZMFBs, the majority of reported ZMFBs have struggled to achieve an areal charge capacity exceeding 20 mAh cm^−2^ (25 mWh cm^−2^) and a limited lifespan (<100 cycles with accumulated capacity < 2000 mAh cm^−2^). Our strategy of membrane design accomplishes a significant advancement, with a record high areal capacity of 100 mAh cm^−2^ (130.1 mWh cm^−2^) at 20 mA cm^−2^, a long lifespan of more than 200 cycles (423 h) with a high accumulated capacity of 6510 mAh cm^−2^ at 30 mA cm^−2^, as well as a demonstration of high current densities to 100 mA cm^−2^ at 30 mAh cm^−2^. Compared to other reported ZFBs, our approach demonstrates comprehensive cost‐performance competitiveness. The inherent low cost of electrolytes (≈$5.17 kWh**^−^** ^1^ at 100 mAh cm^−2^) provides ZMFBs with a cost advantage over other ZFBs, which range from $15 to $353.55 kWh**^−^** ^1^ (see Figure S15 and Table S5, Supporting Information, for more details). Thanks to the effective DcoPZ, it further achieves a cost reduction of 77%, compared to the conventional Nafion membranes of ≈$500 m**^−^** ^2^ commonly applied in ZFBs. More importantly, the near‐neutral battery systems are environmentally safe and eco‐friendly. Building on this foundation, our future development will focus on continuous refinement for enhanced reliability in critical operating conditions while maintaining cost‐performance merits for sustainable energy storage.

## 6 Conclusion

We reported a membrane design strategy to achieve low‐cost and high‐energy‐density ZMFBs for energy storage, by constructing membranes for proton and zinc ions regulation to simultaneously relieve problems of proton crossover in posolytes and dendrite formation in negolytes. In the strategy, the surface‐enriched charge networks, which are created by Zn^2+^ crosslinked on a porous robust substrate, could function in discriminating proton (H^+^) from the conduction ion (K^+^), leveraging the larger ionic potential of protons. It not only markedly barriers proton dehydration from entering the membrane but also imposes an additional retention effect via stronger interactions of protons with nitrogen‐containing groups within the crosslinked domain. In the meantime, the surface‐enriched positive charge induces zinc‐oriented growth by facilitating Zn^2+^ interfacial distribution uniformity in electrode‐adjacent interfacial zones, suppressing dendrite formation. The synergistic strategy significantly improves the performance of near‐neutral ZMFBs, reaching a record high areal capacity of 100 mAh cm^−2^ (130.1 mWh cm^−2^) at 20 mA cm^−2^, a high accumulated capacity of 6510 mAh cm^−2^ at 30 mA cm^−2^, together with a potential demonstration of the current density high to 100 mA cm^−2^ at 30 mAh cm^−2^, representing one of the most stable ZMFBs reported. In addition, the designed membranes save costs by 77% without compromising competitive performance, relative to conventional Nafion membranes (failed at 50 mAh cm^−2^). This cost‐performance superiority, coupled with the environmental friendliness of near‐neutral battery systems, delivers potential in energy storage. Overall, our work provides a feasible and effective strategy to achieve sustainable energy storage technology with low cost and high energy density.

## 7 Experimental Section

### Materials

Polyacrylonitrile (PAN, Mw = 150 000) and manganese dioxide (MnO_2_, 99%) were received from Meryer. Potassium chloride (KCl, 99%), zinc acetate (Zn(CH_3_COO)_2_, 99%), and manganese acetate tetrahydrate (Mn(CH_3_COO)_2_·4H_2_O, 99%) were purchased from Aladdin. PAN Hydrochloric acid (HCl), *N*, *N*‐dimethylformamide (DMF), *N*, *N*‐dimethylacetamide (DMAc), 1‐methyl‐2‐pyrrolidinone (NMP), and potassium iodide (KI, ≥99%) were purchased from Sigma‐Aldrich. All chemicals were used as received without further purification.

### Membrane Fabrication

The fabrication process began with the preparation of a PAN casting solution (2 wt%). To ensure homogeneity, a specified amount of PAN was added to DMAc and stirred overnight. Then, the dissolved casting solution stood for at least 12 h to remove the bubbles. Subsequently, the casting solution was cast on a pristine porous substrate with a blade. The membranes were treated at 60 °C, washed, and stored in water, resulting in the DcoP membranes. To prepare DcoPZ, the PAN casting solution (2 wt%) containing zinc acetate (3 wt%) was prepared following the same fabrication method. The detailed membrane fabrication method is illustrated in Figure S1 (Supporting Information).

### Membrane Characterizations

The cross‐sectional and surface morphology of membranes was characterized by SEM (Quanta 400F) after fracturing them in liquid nitrogen and coating them with platinum, operating at 15 kV. The top‐surface chemical analysis of membranes was determined by the FTIR spectrometer (Thermo Fisher Nicolet iS50) with a scan range from 1000 to 4000 cm^−1^, XPS (Escalab 250Xi) with the binding energy range of 0–1400 eV using an Al Kα 1486.6 eV X‐ray source, The membrane thermal stability was characterized via the thermal gravimetric analyzer (TGA, HITACHI STA200). The element analysis of membranes was obtained from the ICP‐MS (Agilent 720ES(OES)). The static contact angle on the top surface of the membranes was measured by the contact angle tester (Lauda Scientific LSA100). The mechanical properties of the membranes were evaluated by the Universal Testing Machine (CMT5105). The zeta potentials of membranes were measured by a Surface Zeta Potential Analyser (Anton Paar surpass). XRD of deposited zinc on carbon felt was obtained from the Rigaku Smart Lab diffractometer (Cu Kα radiation). The concentration of I_3_^−^ was measured by SEC2000 UV–Visible Spectrophotometer (ALS Co., Ltd.).

### The Transmembrane Ion Permeability Test

The permeability of H⁺ ions was measured using a customized H‐type diffusion cell with an effective area of a circle (diameter of 1.5 cm). The left chamber of the diffusion cell was filled with HCl (40 mL, 1 mol L^−1^), while the right chamber was filled with ultrapure water (40 mL). The concentration of H^+^ ions in the right chamber was determined at regular intervals by a pH meter (Mettler Toledo). The ion concentration was calculated using the following equation:^[^ ^48^ ^]^

(1) $$C=\frac{k}{\Lambda_{m}}$$

where *κ* is the conductivity of the solution of the diffusion side, *c* is the ion concentration, and Λ_m_ is the molar conductivity of metal chloride from references.^[^ ^49^ ^]^ The *κ* of the diffusion side at a certain time was first measured. Then, the *c* of the diffusion side was calculated by *κ* and Λ_m_. The ion permeation rate (*J*, mol·m^−2^ h^−1^) was calculated based on the *c* and the recorded time (*t*).

The permeability of Zn^2+^ ions was measured with ZnCl_2_ solution, following the same steps, where the concentration of Zn^2+^ ions was measured by ICP‐MS.

### Ion Transport Properties of Membranes

As previously reported,^[^ ^5^ ^]^ the ion conductivity was measured using a VMP3 electrochemical testing unit (Bio‐Logic, France) via cyclic voltammetry. KCl solution (0.01, 0.1, 1, 2 mol L^−1^), and HCl solution (0.01, 0.1 mol L^−1^) were prepared with ultrapure water. The membrane was soaked in the lower‐concentration aqueous solution for at least 6 h before testing.

### Area Resistance of Membranes

The area resistances of the membranes were obtained using the same device as for ion transport properties. The KCl solution (2 mol L^−1^) was prepared with ultrapure water, and the membranes were immersed in this solution overnight before testing. The ion conductance (*G*) was calculated from the slope of the *I*–*V* curve. The area resistance was determined as the difference in resistance of a conductive cell with and without a membrane. Each result was an average value of two parallel experiments.

### The Charge Properties of Membranes

The surface charge density (*σ*, mC m^−2^) of the membrane was calculated according to the Gouy–Chapman equation,^[^ ^50^, ^51^ ^]^ as Equation (1):

(2) $$\left|\sigma \right|=\varepsilon \kappa \xi \frac{\sinh\left(\frac{F\xi }{2RT}\right)}{\frac{F\xi }{2RT}}$$

where ε is permittivity (6.933 × 10^−10^ F m^−1^), $\kappa^{-1}={\left(\frac{\varepsilon RT}{2F^{2}C}\right)}^{1/2}$ is Debye length (nm), ξ is surface zeta potential (mV), *F* is Faraday constant (96 485 C mol^−1^), *R* is gas constant (8.314 J mol^−1^ K^−1^), and *T* is the absolute temperature (298 K). The surface zeta potential of membranes was evaluated in a 2 m KCl solution.

### Battery Performance

The carbon felt was purchased from Dalian Longtian Tech. Co., Ltd. without any modification. For full flow batteries, both the positive and negative electrolyte contains Zn(CH_3_COO)_2_ (1 mol L^−1^), Mn(CH_3_COO)_2_·4H_2_O (1 mol L^−1^), KCl (2 mol L^−1^), and KI (0.1 mol L^−1^), with volumes of 10 mL for the positive side and 10 mL for negative side, respectively. When the charge areal capacity of batteries was 60 mAh cm^−2^, KI (0.15 mol L^−1^) was added to the electrolyte, maintaining a volume of 10 mL for the positive side and 10 mL for the negative side. For charge areal capacities exceeding 60 mAh cm^−2^, KI (0.15 mol L^−1^) was added to the electrolyte, with volumes adjusted to 15 mL for the positive side and 20 mL for the negative side. Each performance was two parallel experiments. The electrolytes were circulated through the electrodes using two pumps. The effective area for ZMFBs was 2 × 2 cm^2^. During the cycling tests under 298.15 K and 1 atm, the battery was charged for different durations according to charge capacity (Land, Wuhan Land Electronic Co., Ltd.). For zinc‐zinc symmetric flow batteries, the electrolyte volume is 5 mL for the positive side and a piece of zinc foil (2 cm × 2 cm × 0.1 mm) was placed in the positive side. Other assembling parameters are the same as the full flow batteries.

### Calculation

The geometry optimization and frequency of components were calculated by DFT at the B3LYP^[^ ^52^ ^]^ functional level with a 6‐31g(d) basis set, as implemented in the Gaussian 09W^[^ ^53^ ^]^ packages. The electronic properties were analyzed by Multifwn.^[^ ^54^, ^55^ ^]^ The solvation free energy (Δ*G*_solv_) of zinc with DMAc, NMP, and DMF was calculated based on the electronic energy with the solvent (*E*_solv_) and without the solvent (*E*_gas_). *E*_solv_ and *E*_gas_ were calculated at M052x/6‐31g(d) level. The implicit universal solvation model based on solute electron density (SMD)^[^ ^56^ ^]^ was applied in all calculations. The complexation energies between different Zn^2+^ ions and various solvation environments were calculated with basis set superposition error (BSSE) correction at B3LYP/6‐31+g(d) calculation level, incorporating atom‐pairwise dispersion correction. The complexation energies were determined using the following equation: Complexation energies = *E*(AB) − *E*(A) − *E*(B) + *E*_BSSE_, where *E*(AB), *E*(A), and *E*(B) refer to the energy of the complex AB, fragment A, and fragment B, respectively. *E*_BSSE_ represents the energy of BSSE. The interaction energies of H^+^, K^+^, and Zn^2+^ ions with PAN polymer were calculated according to the differences between reactants and products, at B3LYP/6‐31+g(d,p) calculation level incorporating atom‐pairwise dispersion correction. Based on references,^[^ ^57^ ^]^ a simplified PAN unit with no chirality was used as a model component representing PAN polymer. A two‐dimensional model for a zinc‐manganese flow battery equipped with a D or DcoPZ was employed to simulate the ion distribution at the membrane–electrode interface by the finite‐element method. In the model, the diffusion of ion concentration follows the Nernst–Planck equation. The boundary of the simulation area is set as the current density and voltage control boundary. The current density is set to a nonuniform form. Under the action of an applied voltage, ions in the electrolyte undergo migration and diffusion. The temperature is set at 293.15 K. The concentrations for Zn^2+^ and Cl^−^ are 1 and 2 mol L^−1^, respectively. The diffusion coefficients for Zn^2+^ and Cl^−^ are 7.2 × 10^−6^ and 2.03 × 10^−5^ cm^2^ s^−1^, respectively.^[^ ^49^ ^]^ The transient solution process is based on the PARDISO solver, and the relative tolerance and tolerance factor are set to 10^−8^ and 0.005, respectively.

For molecular dynamics simulation, a polymer membrane model was constructed, consisting of 40 polymer chains, each containing 20 monomer units, with an overall membrane thickness of ≈5 nm. On the right side of the membrane, a mixed electrolyte solution containing Zn(CH_3_COO)_2_ (1 mol L^−1^), Mn(CH_3_COO)_2_·4H_2_O (1 mol L^−1^), KCl (2 mol L^−1^), and HCOOH (0.5 mol L^−1^) was introduced, while the left side was filled with pure water. Water molecules were modeled using the SPC/E model, and the interactions within the polymer and electrolytes were described by the OPLS‐AA force field. Prior to the production runs, the system was first subjected to energy minimization using the conjugate gradient method to eliminate unfavorable atomic configurations and obtain a stable initial structure. Subsequently, molecular dynamics simulations were performed in the canonical ensemble (*NVT*) at 300 K for 10 ns to further equilibrate the system. The temperature was maintained using a Nosé–Hoover thermostat, and long‐range electrostatic interactions were calculated using the particle–particle–particle–mesh (PPPM) method with a cutoff distance of 10 Å. All simulations were carried out using the LAMMPS software package.

The formula for the MSD is given by:

(3) $$MSD\left(t\right)=\frac{1}{N}\sum_{i=1}^{N}|r_{i}\left(t\right)-r_{i}\left(0\right){|}^{2}$$

where *N* is the total number of particles, *r_i_* (*t*) is the position vector of particle 𝑖 at time 𝑡, and *r*_0_(*t*) is the initial position vector of particle 𝑖 at time 𝑡 = 0.

The diffusion coefficient is typically calculated based on the MSD of molecules during molecular dynamics simulations, using Einstein's diffusion equation:

(4) $$D_{s}=\frac{1}{2d}\underset{t\to \infty }{lim}\frac{d}{dt}\left\langle |r\left(t\right)-r\left(0\right){|}^{2}\right\rangle$$

where the term in braces is the ensemble average of the MSD of the compound.

## Conflict of Interest

The authors declare no conflict of interest.

## Supporting information



Supporting Information

## Data Availability

The data that support the findings of this study are available in the supplementary material of this article.
