# Supplementary material for: Ion‐Regulating Membranes with Surface‐Enriched Charge Networks Enabling Stable Zinc‐Manganese Flow Batteries
Source: Adv Mater. 2025 Dec 12;38(9):e17473. doi: 10.1002/adma.202517473 (PMC12902590; doi:10.1002/adma.202517473)
Supplement: Supplementary file 1 — Supporting Information [file ADMA-38-e17473-s001.pdf]

ADVANCED MATERIALS

Supporting Information

for *Adv. Mater.*, DOI 10.1002/adma.202517473

Ion-Regulating Membranes with Surface-Enriched Charge Networks Enabling Stable Zinc-Manganese Flow Batteries

*Jine Wu, Jiafeng Lei and Yi-Chun Lu**

Supporting Information

Ion-regulating membranes with surface-enriched charge networks enabling stable zinc-manganese flow batteries

*Jine Wu[1], Jiafeng Lei[1], and Yi-Chun Lu[1]**

Supplementary Tables 1-5

Table S1 The properties of ions.

Ions	Ionic radius [Å][1-2]	Ionic potential Z^2/r [nm^{-1}]	Hydrated radius R [Å][1-2]	Hydrated Ionic potential Z^2/R [nm^{-1}]	Hydration free energy [kJ mol^{-1}][3]
H^+	1.15	8.70	2.80	3.57	–1049
K^+	1.49	6.71	3.31	3.02	–295
Cl^-	1.81	5.52	3.32	3.01	–340
Zn^{2+}	0.74	54.05	4.30	9.30	–1953

Table S2 The Hansen solubility parameters of PAN in DMF, DMAc, and NMP.

	δ Hansen solubility parameters [(MPa)$^{0.5}$][4]	△δ [(MPa)$^{0.5}$]
PAN	26	/
DMF	24.8	1.2
NMP	22.9	3.1

DMAc	22.1	3.9

Table S3 The ion permeation rate (J) of zinc ions in the permeation test.

Membranes	Zn^{2+} permeation rate [J, mol m^{-2} h^{-1}]	H^+ permeation rate [J, mol m^{-2} h^{-1}]
D	0.270 ± 0.055	2.227 ± 0.027
DcoPZ	0.182 ± 0.013	1.269 ± 0.117

Table S4 Performance comparison for zinc-manganese flow batteries reported recently.

ref	Current density [mA cm^{-2}]	Areal capacity [mAh cm^{-2}]	Cycle numbers	Cycle hours	Energy density [mWh cm^{-2}]	pH of systems
1[5]	20	60	27	162	84.15	near neutral, flow
2[6]	30	2	1800	2400.117	1.95	near neutral, static
3[7]	2	1	200	200	3.9	near neutral, static
4[8]	40	13.3	100	66.5	21.28	near neutral, flow
5[9]	20	5	100	50	9.6	near neutral, flow
6[10]	20	40	60	240	64	near neutral, flow
7[11]	20	18	170	306	36.9	Acid, static
8[12]	1.33	0.33	200	40	0.627	Acid, static
9[13]	30	65.6	26	114	129.8	Alkaline, flow
10[14]	20	30	27	81	54.64	Hybrid, flow
11[15]	9.5	20.4	50	224	25.5	near neutral, static
12[16]	0.2	1	53	N/A	1.44	near neutral, static
13[17]	20	20	100	200	1.91	Acid, flow
This work	20	60	66	365	76.5	near neutral, flow
This work	15	90	33	420	111.7	near neutral, flow
This work	15	100	29	321	130.1	near neutral, flow

Table S5 Cost calculation

Cm ($ m^{-2}) is the cost of membranes.[18] The cost of electrolyte (*Ce*, $ kWh^{-1}) based on

the achieved capacity is calculated as below.

$$Ce = (Ue \times \frac{c_1}{c_2}) \times \frac{1}{V}$$

Ce ($ kWh^{-1}) refers to the cost of the electrolyte per kWh, *Ue* ($ Ah^{-1}) is the cost of the electrolyte per Ah based on the achieved capacity. c_1 is the actual volumetric capacity, and c_2 is the theoretical volumetric capacity. *V* is the cell voltage. *Ur* ($ Ah^{-1}) is the cost of supporting electrolyte.

Chemical	Price [$ kg^{-1}]	*Ue* [$ Ah^{-1}]
$Mn(Ac)_2 \cdot 4H_2O$	0.24[a)]	0.00082
$Zn(Ac)_2$	0.30[19]	0.00137
KCl	0.26[20]	0.00072
KI	2.20[21]	0.00102

a) Quoted from Jiangsu Longcheng Fine Chemical Co., Ltd. on Alibaba.

Supplementary Figures. 1-12

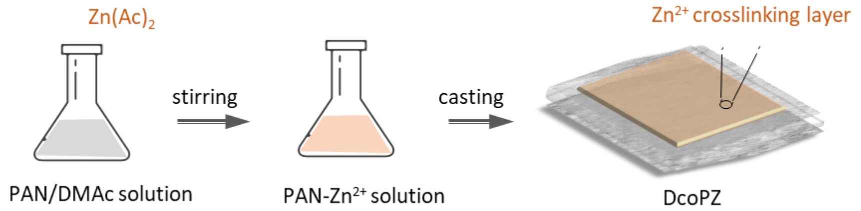

Figure S1 The preparation process of ion selective membranes with Zn^{2+}-crosslinking network designed for zinc-manganese flow batteries.

To prepare membranes, Zn salts were introduced into the organic solution of polyacrylonitrile (PAN), which was cast onto the porous substrate (D) to form the membranes (DcoPZ). Crucially, adequate stirring time and an appropriate organic solution are identified as critical factors in the generation of the crosslinking network (**Table S2 and Figure S2–S5** for further details and DFT calculation). The optimal stirring time facilitates the formation of the Zn^{2+}-crosslinking network while avoiding excessively increasing the areal resistance of membranes. The organic solvent affects the swelling of PAN polymer and the solvation of Zn^{2+} ions, thereby influencing the coordination of Zn^{2+} ions with PAN. In this regard, the membrane fabrication parameters, including stirring time and types of organic solution, were optimized to 24 hours and *N*, *N*-Dimethylacetamide (DMAc), respectively.

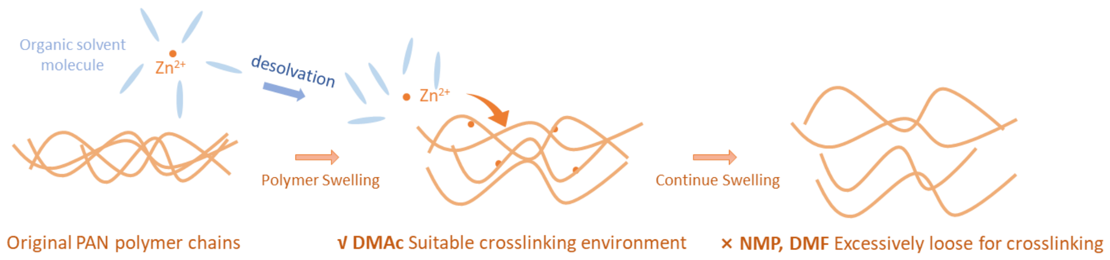

Figure S2 The process of Zn^{2+} ions coordinated with PAN. In this process, the PAN polymer swells in the presence of an organic solvent. The Zn^{2+} ions dissolve in the organic solvents and are subsequently connected with the swollen PAN polymer chains in the organic solvent.

When PAN-Zn^{2+} forms, it is found that DMAc is the preferred organic solvent over DMF and NMP. As shown in **Figure S3**, membranes fabricated in DMAc (DcoPZ-DMAc) enable zinc-manganese flow batteries to operate stably. However, membranes fabricated in DMF (DcoPZ-DMF) exhibited poor stability and were pierced by zinc dendrites. Moreover, the membranes fabricated in NMP (DcoPZ-NMP) showed such high resistance that the batteries assembled with them reached the cut-off voltage at the beginning of the charging process.

It is speculated that the choice of organic solvent affects two aspects. Firstly, the interaction of Zn^{2+} ions with the solvent. The DFT calculation (**Figure S4**) indicates that Zn^{2+} ions exhibit the highest solubility in DMAc, as evidenced by the largest solvation free energy (ΔGsolv) among the organic solvents investigated. Additionally, Zn^{2+} ions have relatively low complexation energy with DMAc, making it easier for them to desolvate when coordinating with PAN polymer chains. Secondly, the formation of PAN-Zn^{2+} is affected by the state of PAN polymers swollen in the organic solvent. According to the Hansen solubility parameters listed in **Table S2**, DMAc has the greatest difference in solubility parameters compared to PAN, resulting in the slowest dissolution rate of PAN in DMAc. This prevents over-swelling and provides an optimal environment for the coordination of Zn^{2+} ions with PAN.

In summary, DMAc is the optimal organic solvent for effectively coordinating Zn^{2+} ions with PAN to generate a continuous Zn^{2+} cross-linked layer, outperforming DMF and NMP.

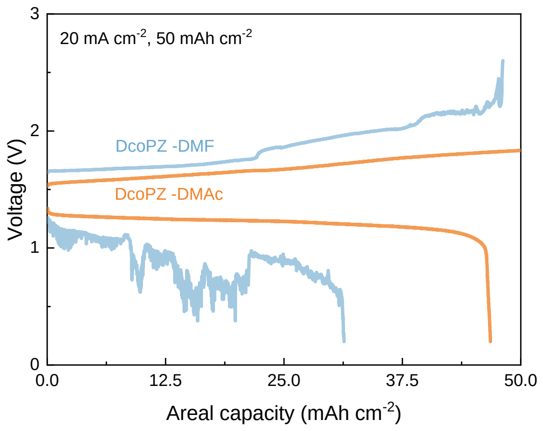

Figure S3 Voltage profiles of ZMFBs assembled with DcoPZ-DMF, DcoPZ-DMAc, and DcoPZ-NMP membranes (20 mA cm^{-2}, 50 mAh cm^{-2}).

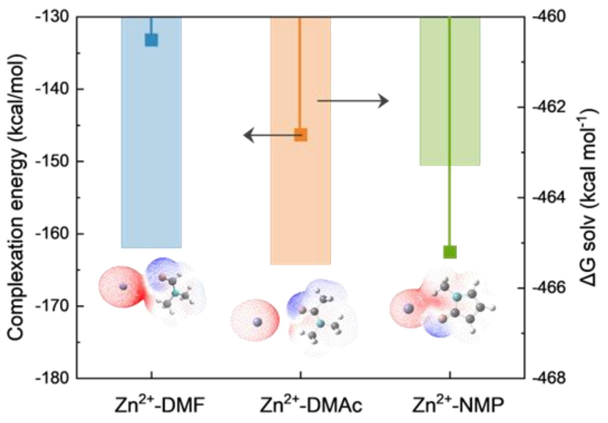

Figure S4 DFT calculation of complexation energy and the solvation free energy (ΔGsolv) of Zn^{2+} ions with different organic solvents (DMF, DMAc, and NMP). The inserts illustrate the complexation of Zn^{2+} ions with DMF, DMAc, and NMP.

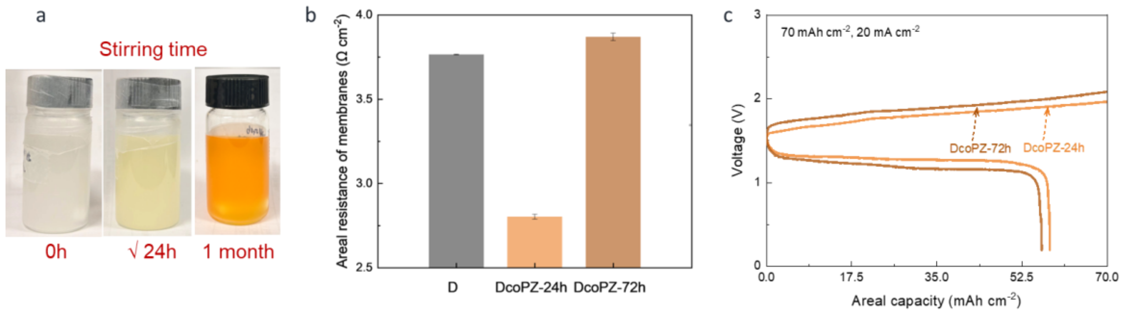

Figure S5 Optimization of reaction time for membrane fabrication. (a) Optical photographs of the PAN-Zn^{2+} solution after stirring for 0 hours, 24 hours, and 1 month. (b) Areal resistance of membranes prepared from the PAN-Zn^{2+} solution at different stirring times. (c) Voltage profiles of zinc-manganese flow batteries assembled with DcoPZ-24h and DcoPZ-72h membranes (20 mA cm^{-2}, 70 mAh cm^{-2}).

When zinc acetate was added to the PAN/DMAc solution, the color of the solution gradually turned orange with the extension of stirring time. The optimal stirring time was determined to be 24 hours, which promotes the formation of the Zn^{2+}-crosslinking network without significantly increasing the areal resistance of membranes (**Figure S5b**). Excessive crosslinking in DcoPZ-72h leads to higher areal resistance, resulting in larger overpotential in the batteries (**Figure S5c)**.

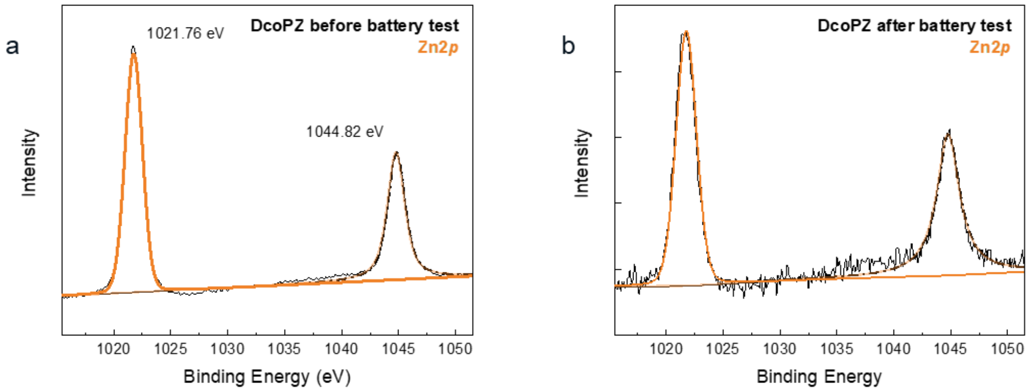

Figure S6 The Zn 2p spectra of XPS spectroscopy on DcoPZ (a) before and (b) after battery test.

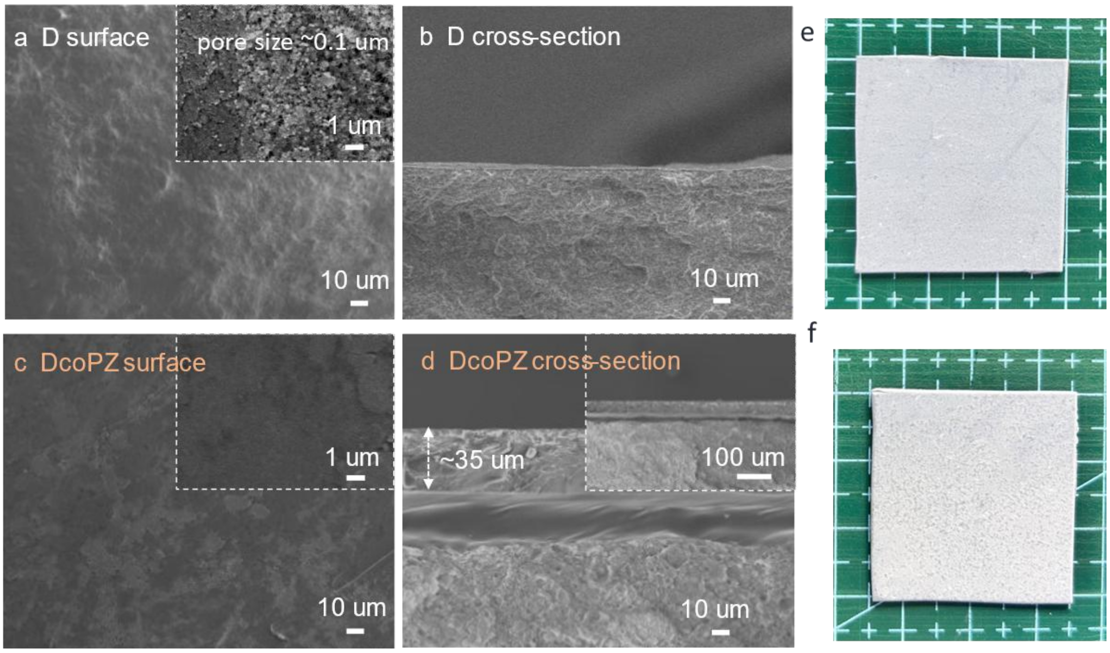

Figure S7 The morphology of membranes. The scanning electron microscopy (SEM) images of porous substrate membranes (D) (a) surface (b) cross-section, and DcoPZ membranes (c) surface (d) cross-section. Photos of one piece of membranes (e) D and (f) DcoPZ.

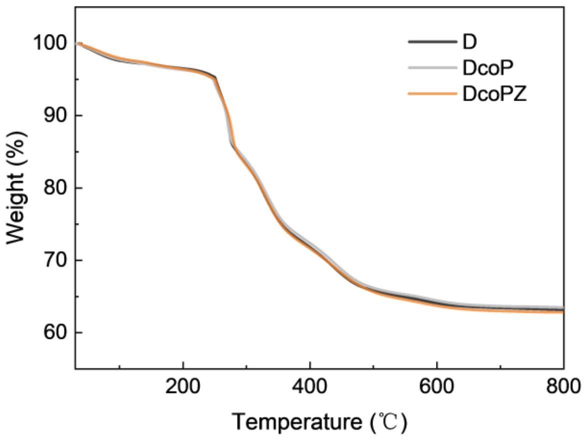

Figure S8 The thermal gravimetric analysis (TGA) of the pristine D, DcoP, and DcoPZ membranes.

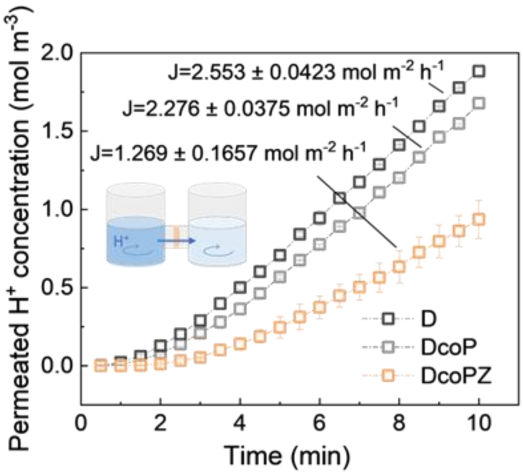

Figure S9 The transmembrane H^+ ions permeation test. The inset is an illustration of the test device.

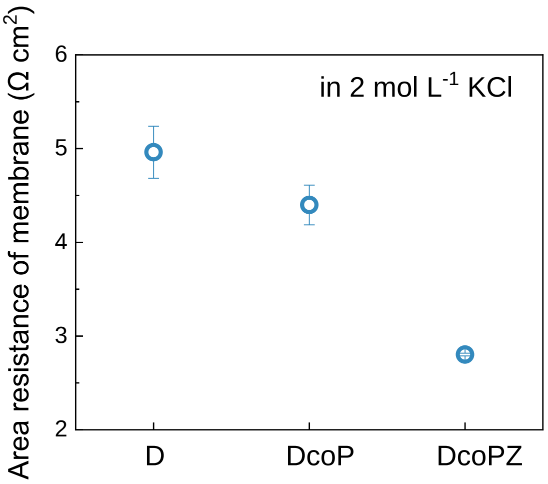

Figure S10 The areal resistance of membranes in 2M KCl. The inset is the contact angle of DcoPZ with 2M KCl. Error bars, mean ± standard deviation (s.d.).

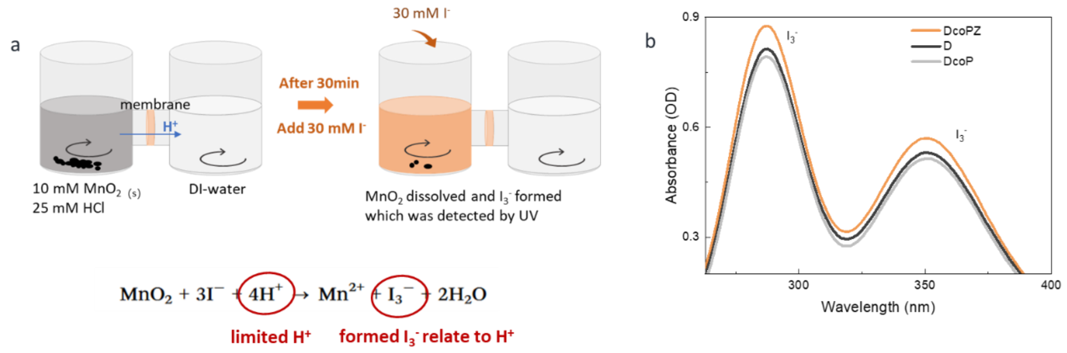

Figure S11 The effect of H^+ ions crossover to the reversibility of MnO_2/Mn^{2+} reaction. (a) The demonstration of the homemade device. (b) The UV-vis spectra of the formed I_3^- related to the remaining H^+ ions in the left side of the H-cell.

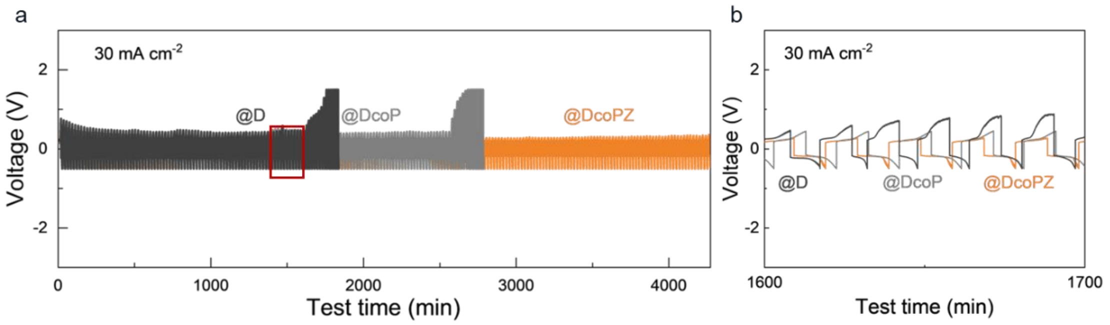

Figure S12 The zinc-zinc symmetric flow batteries were assembled with D, DcoP, and DcoPZ membranes. The voltage profile highlighted in the red box in (a) is enlarged in (b).

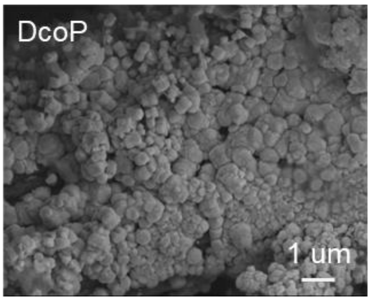

Figure S13 The morphology of zinc deposited on carbon felt observed via SEM at the end of batteries charged and assembled with DcoP (20 mA cm^{-2}, 60 mAh cm^{-2})

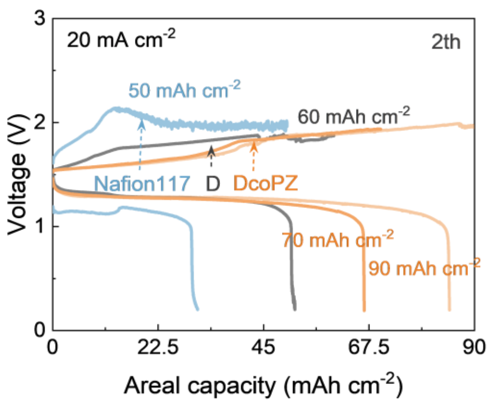

Figure S14 Voltage profiles of batteries assembled with commercial Nafion membranes (50 mAh cm^{-2}), porous substrate D (60 mAh cm^{-2}), and designed DcoPZ (70 mAh cm^{-2}, 90 mAh cm^{-2}) at a current density of 20 mA cm^{-2}.

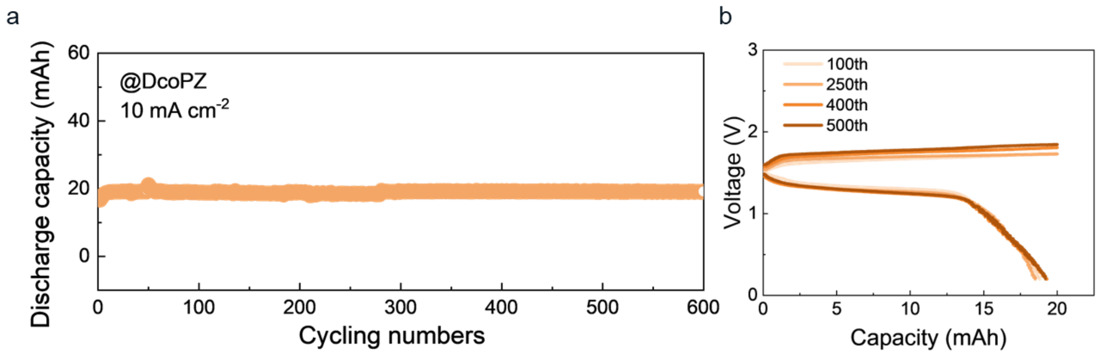

Figure S15 (a) The long-term cycling performance and (b) voltage profiles of batteries assembled with the designed DcoPZ.

Systems	Battery Performance Indicators					Cost Indicators		Ref.
	Areal capacity (mAh cm^{-2})	Current density (mA cm^{-2})	Cycle life (hours)	Accumulate charged capacity* (mAh cm^{-2})	Energy density** (mWh cm^{-2})	Electrolyte cost ($/kWh)	Membrane cost ($/m^2)	
Zn-Br	14.28	20	85.68	856.80	24.28	74***	500	1
Zn-I	7.50	30	175	2625	9	353.55	10	2
Zn-Fe (neutral)	40.21	40	201	4021	56.30	15	50	3
Zn-Fe (alkaline)	17.95	200	35.90	3591.95	31.25	40	12.31	4
Zn-Mn (alkaline)	65.60	30	114	1705.60	129.88	/	500	5
Zn-Mn (acid)	20	20	200	2000	38.20	/	500	6
Zn-Mn (neutral)	1	2	200	200	1.95	/	/	7****
	1	0.20	/	53	1.44	/	/	8****
	13.30	40	66.50	1330	21.28	/	/	9
	20.40	9.50	224	1020	25.50	/	/	10
	60	20	162	1620	84.15	/	500	11
Zn-Mn (neutral) This work	30	30	423	6510	35.65	11.50	31.52	
	60	20	365	3780	76.50	5.75		
	90	15	420	2790	111.74	5.75		
	100	15	321	2600	130.05	5.17		

* Estimated based on the charging capacity with CE=100% and no capacity decay.
** The capacity from mediator has been eliminated when calculating energy density in this work.
*** Not mentioned in reference, the value was obtained from refence12. **** static cells

Figure S16 Performance and cost comparison of ZMFBs in this work with other standard zinc-based flow batteries. The representative Zn-Mn static cells (ref 7 and ref 8) are also included here for comprehensive comparison. References: 1[22], 2[23], 3[20], 4[24], 5[13],6[17], 7[7], 8[16], 9[8], 10[15], 11[5], 12[25]. *Estimated based on the charging capacity with CE=100% and no capacity decay. **The capacity from the mediator has been eliminated when calculating energy density in this work. ***Not mentioned in reference, the value was obtained from reference12. ****static cells.

Figure S16 employed a color-coding system whereby each indicator is represented by the same color, with the shade of the color indicating the value of the corresponding indicator. In particular, a dark color of the performance indicators and cost indicators denoted superior performance and lower cost. Please refer to **Table S5** for cost calculation.

References

[1] B. Tansel, *Significance of thermodynamic and physical characteristics on permeation of ions during membrane separation: Hydrated radius, hydration free energy and viscous effects*, *Sep. Purif. Technol.* **2012**, 86, 119.

[2] A. G. Volkov, S. Paula, D. W. Deamer, *Two mechanisms of permeation of small neutral molecules and hydrated ions across phospholipid bilayers*, *Bioelectrochem. Bioenerg.* **1997**, 42, 153.

[3] H. Binder, O. Zschornig, *The effect of metal cations on the phase behavior and hydration characteristics of phospholipid membranes*, *Chem. Phys. Lipids* **2002**, 115, 39.

[4] J. Carper, *The CRC Handbook of Chemistry and Physics*, *Libr J* **1999**, 124, 192.

[5] J. Lei, Y. Yao, Z. Wang, Y.-C. Lu, *Towards high-areal-capacity aqueous zinc–manganese batteries: promoting MnO2 dissolution by redox mediators*, *Energy Environ. Sci.* **2021**, 14, 4418.

[6] D. Chao, W. Zhou, C. Ye, Q. Zhang, Y. Chen, L. Gu, K. Davey, S.-Z. Qiao, *An Electrolytic Zn–MnO2 Battery for High-Voltage and Scalable Energy Storage*, *Angew. Chem. Int. Ed.* **2019**, 58, 7823.

[7] Y. Deng, H. Wang, M. Fan, B. Zhan, L. J. Zuo, C. Chen, L. Yan, *Nanomicellar Electrolyte To Control Release Ions and Reconstruct Hydrogen Bonding Network for Ultrastable High-Energy-Density Zn-Mn Battery*, *J. Am. Chem. Soc.* **2023**, 145, 20109.

[8] C. X. Xie, T. Y. Li, C. Z. Deng, Y. Song, H. M. Zhang, X. F. Li, *A highly reversible neutral zinc/manganese battery for stationary energy storage*, *Energy Environ. Sci.* **2020**, 13, 135.

[9] R. p. Naresh, K. Mariyappan, D. Dixon, M. Ulaganathan, P. Ragupathy, *Investigations on New Electrolyte Composition and Modified Membrane for High Voltage Zinc−Manganese Hybrid Redox Flow Batteries*, *Batteries Supercaps* **2021**, 4, 1464.

[10] Y. Liu, C. Xie, X. Li, *Carbon Nanotube Network Induces Porous Deposited MnO(2) for High-Areal Capacity Zn/Mn Batteries*, *Small* **2024**, 20, e2402026.

[11] Y. F. Cui, Z. B. Zhuang, Z. L. Xie, R. F. Cao, Q. Hao, N. Zhang, W. Q. Liu, Y. H. Zhu, G. Huang, *High-Energy and Long-Lived Zn-MnO(2) Battery Enabled by a Hydrophobic-Ion-Conducting Membrane*, *ACS Nano* **2022**, 16, 20730.

[12] Y. Yuan, J. Yang, Z. Liu, R. Tan, M. Chuai, J. Sun, Y. Xu, X. Zheng, M. Wang, T. Ahmad, N. Chen, Z. Zhu, K. Li, W. Chen, *A Proton-Barrier Separator Induced via Hofmeister Effect for High-Performance Electrolytic MnO2–Zn Batteries*, *Adv. Energy Mater.* **2022**, 12, 2103705.

[13] W. Z. Xiang, M. H. Yang, M. Ding, X. X. Chen, J. L. Liu, G. M. Zhou, C. K. Jia, G. I. N. Waterhouse, *Alkaline Zn-Mn aqueous flow batteries with ultrahigh voltage and energy density*, *Energy Storage Mater.* **2023**, 61, 102894.

[14] J. Cao, K. Yu, J. Zhang, B. Lu, J. Yu, S. Huang, F. Zhang, *Vanadium-Mediated High Areal Capacity Zinc–Manganese Redox Flow Battery*, *ACS Sustainable Chem. Eng.* **2024**, 12, 6320.

[15] N. Zhang, F. Cheng, J. Liu, L. Wang, X. Long, X. Liu, F. Li, J. Chen, *Rechargeable*

aqueous zinc-manganese dioxide batteries with high energy and power densities, *Nat Commun* **2017**, 8, 405.
[16]H. L. Pan, Y. Y. Shao, P. F. Yan, Y. W. Cheng, K. S. Han, Z. M. Nie, C. M. Wang, J. H. Yang, X. L. Li, P. Bhattacharya, K. T. Mueller, J. Liu, *Reversible aqueous zinc/manganese oxide energy storage from conversion reactions*, *Nat. Energy* **2016**, 1, 16039.
[17]Y. Wang, H. Hong, Z. Wei, D. Li, X. Yang, J. Zhu, P. Li, S. Wang, C. Zhi, *Cation-regulated MnO2 reduction reaction enabling long-term stable zinc–manganese flow batteries with high energy density*, *Energy Environ. Sci.* **2025**, 18, 1524.
[18]L. Qiao, S. Liu, M. Fang, M. Yang, X. Ma, *A Composite Membrane with High Stability and Low Cost Specifically for Iron-Chromium Flow Battery*, *Polymers* **2022**, 14.
[19]J. Lei, Y. Yao, Y. Huang, Y.-C. Lu, *A Highly Reversible Low-Cost Aqueous Sulfur–Manganese Redox Flow Battery*, *ACS Energy Lett.* **2022**, 8, 429.
[20]C. Xie, Y. Duan, W. Xu, H. Zhang, X. Li, *A Low-Cost Neutral Zinc-Iron Flow Battery with High Energy Density for Stationary Energy Storage*, *Angew. Chem. Int. Ed.* **2017**, 56, 14953.
[21]Z. J. Li, Y. C. Lu, *Polysulfide-based redox flow batteries with long life and low levelized cost enabled by charge-reinforced ion-selective membranes*, *Nat. Energy* **2021**, 6, 517.
[22]G. Choi, P. Sullivan, X. L. Lv, W. Li, K. Lee, H. Kong, S. Gessler, J. R. Schmidt, D. Feng, *Soft-hard zwitterionic additives for aqueous halide flow batteries*, *Nature* **2024**, 635, 89.
[23]Z. Wei, Z. Huang, G. Liang, Y. Wang, S. Wang, Y. Yang, T. Hu, C. Zhi, *Starch-mediated colloidal chemistry for highly reversible zinc-based polyiodide redox flow batteries*, *Nat Commun* **2024**, 15, 3841.
[24]Z. Z. Yuan, L. X. Liang, Q. Dai, T. Y. Li, Q. L. Song, H. M. Zhang, G. J. Hou, X. F. Li, *Low-cost hydrocarbon membrane enables commercial-scale flow batteries for long-duration energy storage*, *Joule* **2022**, 6, 884.
[25]G. P. Rajarathnam, 2016.
